# Model Checking Temporal Logic Formulas Using Sticker Automata

**DOI:** 10.1155/2017/7941845

**Published:** 2017-09-28

**Authors:** Weijun Zhu, Changwei Feng, Huanmei Wu

**Affiliations:** ^1^School of Information Engineering, Zhengzhou University, Zhengzhou 450001, China; ^2^The Second Affiliated Hospital of Zhengzhou University, Zhengzhou 450001, China; ^3^School of Informatics and Computing, Indiana University-Purdue University Indianapolis, Indianapolis, IN, USA

## Abstract

As an important complex problem, the temporal logic model checking problem is still far from being fully resolved under the circumstance of DNA computing, especially Computation Tree Logic (CTL), Interval Temporal Logic (ITL), and Projection Temporal Logic (PTL), because there is still a lack of approaches for DNA model checking. To address this challenge, a model checking method is proposed for checking the basic formulas in the above three temporal logic types with DNA molecules. First, one-type single-stranded DNA molecules are employed to encode the Finite State Automaton (FSA) model of the given basic formula so that a sticker automaton is obtained. On the other hand, other single-stranded DNA molecules are employed to encode the given system model so that the input strings of the sticker automaton are obtained. Next, a series of biochemical reactions are conducted between the above two types of single-stranded DNA molecules. It can then be decided whether the system satisfies the formula or not. As a result, we have developed a DNA-based approach for checking all the basic formulas of CTL, ITL, and PTL. The simulated results demonstrate the effectiveness of the new method.

## 1. Introduction

Differing from an electronic computer, a DNA computer uses DNA molecules as the carrier of computation. In 1994, a Turing Award winner Professor Adleman published an article in 〈Science〉 that solved a small-scale Hamiltonian path problem with a DNA experiment [1], which is regarded as the pioneering work in the field of DNA computing. As DNA computing has a huge advantage for parallel processing, this technique was subsequently advanced rapidly. Many models and approaches based on DNA computing have been developed to solve some complex computational problems, especially the famous NP-hard problems and PSPACE-hard ones. For example, Lipton published an article in 〈Science〉 that improved Adleman's idea for the SAT problem [2]. Ouyang et al. published an article in 〈Science〉 that presented a DNA-computing-based model for solving the maximal clique problem [3]. Benenson et al. published an article in 〈Nature〉 that solved an automata problem of two states and two characters using the autonomous DNA computing technique [4].

Many other DNA models have been constructed, such as the restricted model [5], the sticker system [6], the length-encoding model [7], the sticker automaton model [8], the DNA Turing machine model [9], the nonenumerative DNA model [10], the giant-magneto-resistance-based DNA model [11], the logical DNA molecular model [12], and the logical nanomolecular model [13]. And a series of methods based on nonautonomous or self-assembling are proposed for solving various complex computational problems, including the Nondeterministic Polynomial (NP) ones. For example, there are methods proposed for the maximum clique problem [14, 15], the vertex coloring one [10, 16], the SAT one [11], the *N* queen one [17], the maximum matching one [18], the minimum vertex cover one [19], the minimum and exact cover one [20], the subset-sum one [21], the classical Ramsey number one [22], the spatial cluster analysis [23, 24], and the knapsack [25].

On the one hand, some problems in computer science can be solved by applying the techniques based on biochemical reactions in test tubes, nanodevices, or molecular self-assembly [1, 26–28]. On the other hand, due to the excellent information processing mechanism and the huge parallelism, some living cells can also be employed to perform some computations. The site-specific DNA recombinase Hin, which can mediate inversion of DNA segments that represent variables, was used to produce the solution. In this model, each cell can produce and examine a solution of satisfiability problem. As a result, billions of cells can explore billions of possible solutions [29]. In this way, Professor Chen et al. constructed a cellular computing model [29] to solve the satisfiability problem. In addition, a conditional learning system in* Escherichia coli* was built to identify the “bad man” signal with the help of the “learning” signal. It is a useful attempt to construct the artificial intelligent system using some molecular biological techniques.

One of the key differences between computer and other computing tools is the universality. Professor Xu constructed a mathematical model called “probe machine” for the general DNA computer [30]. By integrating the storage system, operation system, detection system, and control system into a whole, a real general DNA computer was gradually obtained, which was the “Zhongzhou DNA computer” [30]. A probe machine is a nine-tuples consisting of data library, probe library, data controller, probe controller, probe operation, computing platform, detector, true solution storage, and residue collector [15]. It is a universal DNA computing model which can be realized in biology. And a Turing machine is just a “special case” of a probe machine [15]. This significant progress has raised the practical importance of the researches on DNA computing.

More studies on DNA computing have been conducted for the last three years. Some of the major studies are summarized as follows: (1) aiming to deal with some inherent flaws of DNA computing, such as adaptability [31] and instability [32]; (2) employing DNAs to realize some basic computing components and/or techniques, such as data storage [33], database operations [34], odd parity checker [35], half adder [36], encryption [37], and data hiding [38]; (3) utilizing DNAs to address some problems in real world, such as the inverse kinematics redundancy problem of six-degree-of-freedom humanoid robot arms [39], dynamic control of elevator systems [40], and hyperspectral remote sensing data/imagery [41, 42].

Besides the satisfiability problem, model checking (MC) is another important computational problem. These two problems are correlated. The MC proposed by the Turing Award winner Professor Clarke et al. [43] is widely used in the fields of CPU verification [44], network protocol verification, security protocol verification [45], and software verification [46]. MC algorithms answer automatically the question of whether a system satisfies the given property or not. NASA, Intel, IBM, and Motorola are using this technique. The general principles of MC can be given as follows: (i) a system model is constructed with an automaton; (ii) a property which the system should satisfy is described by a temporal logic formula; and (iii) if an automaton is a model of the formula, the system model satisfies the property; otherwise, the system does not satisfy the property.

In order to describe the different temporal properties, some different temporal logic types have been proposed. For instance, Linear Temporal Logic (LTL) was introduced into computer science to express the linear properties by the Turing Award winner Professor Pnueli [47]. Computation Tree Logic (CTL) was proposed to express the branch properties by the Turing Award winner Professor Clarke [48, 49]. Interval Temporal Logic (ITL), Duration Calculus (DC), and Projection Temporal Logic (PTL) were also investigated to express other temporal properties [50–52].

As a complex computational problem, model checking under the circumstance of DNA computing is always a goal for researchers. In 2006, some DNA molecules were applied to conduct CTL model checking for the first time by the Turing Award winner Professor Emerson et al. [53]. However, this method can check only one basic CTL formula, called EF*p*. It is known that there are eight basic formulas in CTL, that is, E*p*U*q*, A*p*U*q*, EF*p*, AF*p*, EG*p*, AG*p*, EX*p*, and AX*p*. It has been a pending and challenging issue to perform model checking for all of the eight basic CTL formulas using DNA computing. As shown in Table 1, there are eight basic formulas for CTL, two for ITL, and one for PTL. Except for the EF*p* formula, all the other ten basic formulas in CTL, ITL, and PTL cannot conduct model checking under the circumstance of DNA computing using the existing methods.

Motivated by it, we proposed a set of DNA-based model checking algorithms. With our new algorithms, all the eleven basic formulas for CTL, ITL, and PTL can undergo model checking via some DNA molecules. Basically, the core model checking problem for the CTL, ITL, and PTL is solved by DNA computing, because every CTL/ITL/PTL formula can be obtained by combining the basic CTL/ITL/PTL formulas recursively. This is the main contribution of this paper.

The rest of this paper is organized as follows.  Section 2 introduces some basic concepts. Our newly proposed algorithms will be described in Section 3. The simulated experiments will be presented in Section 4, which demonstrates that the new algorithms are feasible in molecular biology.  Section 5 provides brief conclusions. The formal definitions of these temporal logic types are given in the Appendix.

## 2. Preliminary

### 2.1. The Basic Formulas in CTL [43]

**Definition 1 .**

Let *p* and *q* be atomic propositions and E*p*U*q*, A*p*U*q*, EF*p*, AF*p*, EG*p*, AG*p*, EX*p*, and AX*p* be the basic CTL formulas. An arbitrary CTL formula can be obtained by recursive combinations of these basic CTL formulas.

An atomic proposition and a basic CTL formula are interpreted on a system model M, and their intuitive meanings are given as follows:

- *p* or *q* is satisfied in a state *s*.
- E*p*U*q* describes the property: there exists at least one path in M, such that *p* is always satisfied until *q* is satisfied.
- A*p*U*q* describes the property: for each path in M, *p* is always satisfied until *q* is satisfied.
- EF*p* describes the property: there exists at least one path in M, such that *p* is eventually satisfied.
- AF*p* describes the property: for each path in M, *p* is eventually satisfied.
- EG*p* describes the property: there exists at least one path in M, such that *p* is always satisfied.
- AG*p* describes the property: for each path in M, *p* is always satisfied.
- EX*p* describes the property: there exists at least one path in M, such that *p* is satisfied in the next state.
- AX*p* describes the property: for each path in M, *p* is satisfied in the next state.

**Figure 1:**
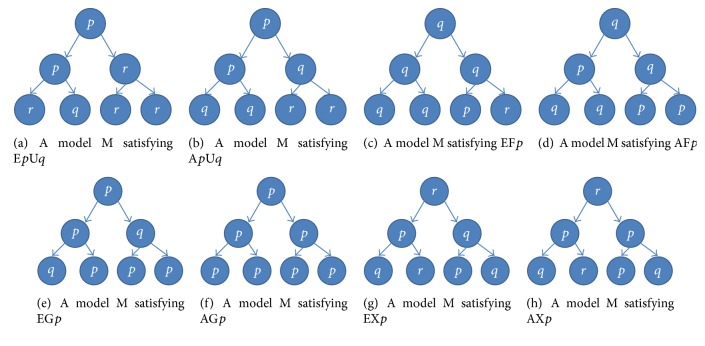
Examples of the basic CTL formulas and their models.

Figure 1 gives some example models which satisfy the eight basic CTL formulas. A circle represents a state, and a letter in a circle represents an atomic proposition which is satisfied in the state. A line segment with an arrow means an edge (i.e., a transition between two states). A state sequence from the root node to a leaf node is called a path. Time passes from top to bottom, and the different branches represent the alternative transitions from the current state to the next one.

For the model M in Figure 1(a), there are four paths. Each path passes through three states at three moments, which forms four sequences of atomic propositions: *ppr*, *ppq*, *prr*, and *prr*. It is noticeable that *ppq*, that is, the second path, satisfies the following property: *p* is always satisfied until *q* is satisfied. In contrast, any other path in M does not satisfy this property. According to the definition of E*p*U*q*, the model M satisfies E*p*U*q*.

For the model M in Figure 1(b), there are also four paths with three states for each path. The four sequences of atomic propositions are: *ppq*, *ppq*, *pqr* and *pqr*. All paths in M satisfy the property: *p* is always satisfied until *q* is satisfied. According to the definition of A*p*U*q*, the model M satisfies A*p*U*q*.

Similarly, for the model M in Figure 1(c), the path *qqp* in M satisfies the property: *p* is eventually satisfied. Thus, the model M satisfies EF*p*. For the model M in Figure 1(d), all four paths, *qpq*, *qpq*, *qqp*, and *qqp*, satisfy the property: *p* is eventually satisfied. Thus, the model satisfies AF*p*. For the model M in Figure 1(e), the path *ppp* in M satisfies the property: *p* is always satisfied. Thus, the model M satisfies EG*p*. For the model M in Figure 1(f), all the four paths, *ppp*, *ppp*, *ppp*, and *ppp*, satisfy the property: *p* is always satisfied, which makes the model M satisfy AG*p*. For the model M in Figure 1(g), the paths *rpq* and *rpr* in M satisfy the property: *p* is satisfied in the next state. Thus, M satisfies EX*p*. For the model M in Figure 1(h), all the four paths, *rpq*, *rpr*, *rpp*, and *rpq*, satisfy the property: *p* is satisfied in the next state. Thus, this M satisfies AX*p*.

Given an arbitrary model M, the challenge is how to use the DNA-computing-based method to determine whether the eight basic CTL formulas are satisfied by M or not.  Section 3.1 will provide our new approach which can check all the eight basic CTL formulas.

### 2.2. The Basic Formulas in LTL [43]

**Definition 2 .**

Let *p* and *q* be atomic propositions and *p*U*q*, F*p*, G*p*, and X*p* be the basic LTL formulas. An arbitrary LTL formula can be obtained by combining recursively some basic LTL formulas. An atomic proposition and a basic LTL formula are interpreted on a path L and a system model M, and their intuitive meanings are given as follows:

- *p* or *q* is satisfied in a state *s*, or not.
- *p*U*q* describes the property: for each path L in M, *p* is always satisfied until *q* is satisfied.
- F*p* describes the property: for each path L in M, *p* is eventually satisfied.
- G*p* describes the property: for each path L in M, *p* is always satisfied.
- X*p* describes the property: for each path L in M, *p* is satisfied in the next state.

For a path L, time passes from left to right, and the system transits from the current state to the next one. For a model M, time passes from top to bottom, and the different branches represent the alternative transitions from the current state to the next one.

**Figure 2:**
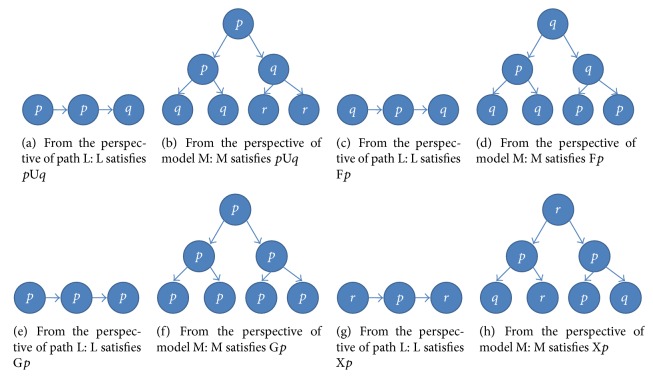
Examples of the basic LTL formulas and their models.

Figure 2 gives one sample M for each basic LTL formula, respectively. The formula *p*U*q* is called the core LTL formula since every basic LTL formula can be expressed by *p*U*q*. Given an arbitrary model M, previous studies have provided approaches on how to use the DNA-computing-based method to determine whether the four basic LTL formulas are satisfied by M or not [54, 55].

### 2.3. The Basic Formulas in ITL [50]

**Definition 3 .**

Let *p*, *q*, *p*_1_, *p*_2_, *q*_1_, and *q*_2_ be atomic propositions and (*p*_1_U*q*_1_); (*p*_2_U*q*_2_) and (*p*U*q*)^*∗*^ be the basic ITL formulas. An arbitrary ITL formula can be obtained by combining recursively some basic ITL formulas. A basic ITL formula is interpreted on a path L and a system model M, and their intuitive meanings are given as follows.

- (*p*_1_U*q*_1_); (*p*_2_U*q*_2_): for each path L in M, the following property holds: prefix subpath (i.e., prefix interval) satisfies the core LTL formula *p*_1_U*q*_1_, and suffix subpath (i.e., suffix interval) satisfies the core LTL formula *p*_2_U*q*_2_.
- (*p*U*q*)^*∗*^: for each path L in M, the following property holds: L circulates in a loop body consisting of a subpath (i.e., a loop body consisting of an interval), and the loop body of interval satisfies the core LTL formula *p*U*q*.

**Figure 3:**
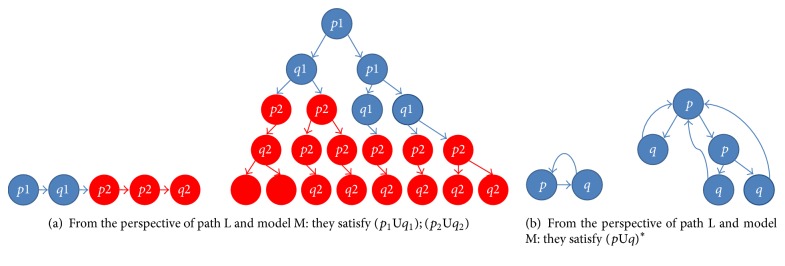
Examples of the basic ITL formulas and their models.

Figure 3 gives an example for each basic ITL formula, respectively. For (*p*_1_U*q*_1_); (*p*_2_U*q*_2_), any path L in M has the following characteristics: L reaches a number of red states after it crosses some blue states. The prefix interval of L is denoted as the state sequence marked in blue, whereas the suffix interval of L is denoted as the state sequence marked in red. An ITL formula is satisfied in such an interval. In Figure 3(a), *p*_1_*q*_1_*p*_2_*p*_2_*q*_2_ is a path satisfying the ITL formula (*p*_1_U*q*_1_); (*p*_2_U*q*_2_). The prefix interval of *p*_1_*q*_1_*p*_2_*p*_2_*q*_2_ satisfies the LTL formula *p*_1_U*q*_1_, whereas the suffix interval of *p*_1_*q*_1_*p*_2_*p*_2_*q*_2_ satisfies the LTL formula *p*_2_U*q*_2_. Similarly, all the paths in M of Figure 3(a) satisfy the ITL formula (*p*_1_U*q*_1_); (*p*_2_U*q*_2_). In Figure 3(b), *pqpq*… is a path satisfying the ITL formula (*p*U*q*)^*∗*^. The loop body consisting of an interval (i.e., *pq*) satisfies the LTL formula *p*U*q*. Similarly, all the paths in M of Figure 3(b) satisfy the ITL formula (*p*U*q*)^*∗*^.

Given an arbitrary model M, we will provide a new solution in Section 3.2 on how to use the DNA-computing-based method to determine whether the two basic ITL formulas are satisfied by M or not.

### 2.4. The Basic Formula in PTL [52]

**Definition 4 .**

Let *p*_1_, *p*_2_, *p*_3_, *q*_1_, *q*_2_, and *q*_3_ be atomic propositions and ((*p*_1_U*q*_1_), (*p*_2_U*q*_2_)) prj (*p*_3_∧X*q*_3_) be the basic PTL formula. An arbitrary PTL formula can be obtained by combining recursively the basic PTL formula. A basic PTL formula is interpreted on a path L and a system model M, and its intuitive meaning is given as follows:

- ((*p*_1_U*q*_1_), (*p*_2_U*q*_2_)) prj (*p*_3_∧X*q*_3_): for each path L in M, the following property holds: (1) the prefix subpath (i.e., the fined-grained prefix interval) satisfies the core LTL formula *p*_1_U*q*_1_, (2) the suffix subpath (i.e., the fined-grained suffix interval) satisfies the core LTL formula *p*_2_U*q*_2_, and (3) the state sequence consisting of the first state in the fine-grained prefix interval and the first state in the fine-grained suffix interval (i.e., the coarse-grained interval) satisfies the LTL formula *p*_3_∧X*q*_3_.

**Figure 4:**
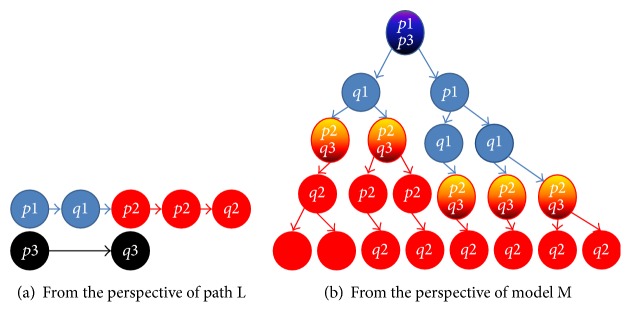
An example of the basic PTL formula ((*p*_1_U*q*_1_), (*p*_2_U*q*_2_)) prj (*p*_3_∧X*q*_3_) and its model.

Figure 4 gives a sample model for the basic PTL formula. For ((*p*_1_U*q*_1_), (*p*_2_U*q*_2_)) prj (*p*_3_∧X*q*_3_), any path L in M has the three intervals: the fined-grained prefix interval is made up of the blue states, the fined-grained suffix interval is made up of the red states, and the coarse-grained interval is made up of the black states. In fact, the difference of the fined-grained intervals and the coarse-grained interval is the different units of time elapse.

Given an arbitrary model M, we will provide a new solution in Section 3.3 on how to use the DNA-computing-based method to determine whether the basic PTL formula is satisfied by M or not.

### 2.5. Finite State Automata and Model Checking

**Definition 5 .**

A Finite State Automaton (FSA) is a five-tuples (Σ, *Q*, *T*, *q*_0_, *F*), where

- Σ is a finite alphabet,
- *Q* is a finite set of states,
- *T* is a finite set of transitions: *T* : *Q* × Σ → *R*(*Q*),
- *q*_0_ ∈ *Q* is an initial state,
- *F*⊆*Q* is a set of acceptance states.

Figure 6 depicts an example for an FSA. This automaton is made up of two states and two transitions. State 0 is an initial state which is pointed at by an arrow without source, whereas state 1 is an acceptance state which is marked by a double circle. The automaton will enter state 0 if *p* is input at state 0, whereas the automaton will enter state 1 if *q* is input at state 0. The string *pq* is an acceptance word, since the automaton will transit from an initial state to an acceptance state if *pq* is input. Similarly, the strings *q*, *ppq*, *pppq*,… are acceptance words too. An acceptance language of an automaton is made up of all of the acceptance words of the automaton. In this example, {*q*, *pq*, *ppq*, *pppq*,…} is the acceptance language of the automaton which is illustrated by Figure 6.

The only difference between the automaton in Figure 6 and the one in Figure 7 is that the atomic propositions in the latter automaton are satisfied in the states rather than in the transitions. Therefore, the latter automaton is called a Label FSA (LFSA).

**Figure 5:**
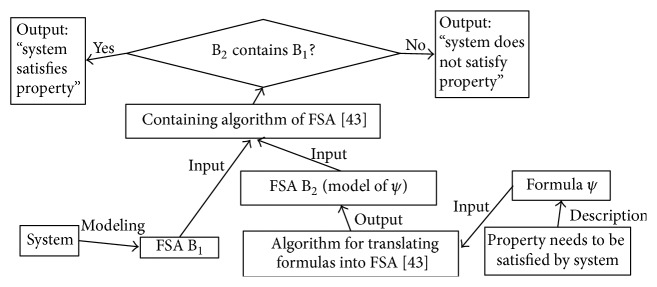
Principle of the model checking algorithms based on classic computing.

**Figure 6:**
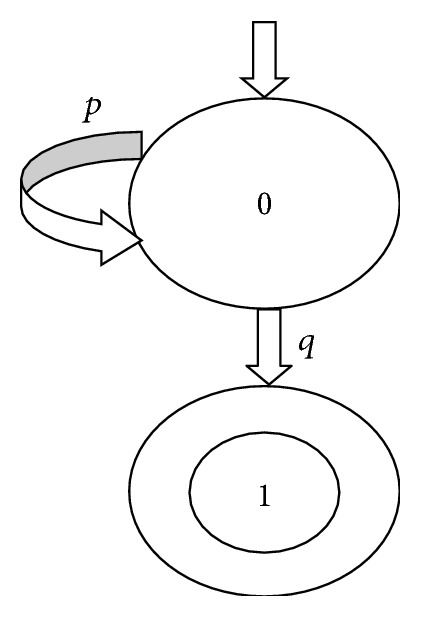
An example on FSA.

In classical computation, the principles of the algorithms for temporal logic model checking can be illustrated by Figure 5. A LFSA, denoted as B_1_, is used to describe some behaviors of a system, whereas an FSA, denoted as B_2_, is employed to construct a model of a temporal logic formula. The model checking algorithm will decide that the system meets the property specified by the formula, if some inclusion relations hold between the two acceptance languages of the two automata.

### 2.6. Sticker Automata and DNA Model Checking

#### 2.6.1. Sticker Automata

As a model of DNA computing, a* sticker automaton* can realize an FSA. Given a DNA strand characterizing an input string and an FSA, the sticker automaton can determine whether or not the string is accepted by the FSA.

M = (Σ, *S*, *T*, *s*_0_, *F*) is an FSA, and every character *a* in the alphabet Σ can be encoded as *C*(*a*). One way of the DNA encoding is as follows [56]:

1. An input string *a*_1_,…, *a*_*n*_ in Σ can be encoded with the single-stranded DNA molecule: 5′ *I*_1_ *X*_0_ ⋯ *X*_*m*_ *C*(*a*_1_) ⋯ *X*_0_ ⋯ *X*_*m*_ *C*(*a*_*n*_) *X*_0_ ⋯ *X*_*m*_ *I*_2_ 3′, where *I*_1_ is an initiator sequence, *X*_0_ ⋯ *X*_*m*_ is a spacer sequence separating *C*(*a*_*i*_), and *I*_2_ is a terminator sequence.
2. A transition *T*(*s*_*i*_, *a*) = *s*_*j*_ is encoded as 3′ $\bar{X_{i+1}\cdots X_{m}}\;\bar{C(a)}\;\bar{X_{0}\cdots X_{j}}$ 5′, where $\bar{X}$ means the Watson-Crick complement (WC for short) of a nucleotide *X* and $\bar{C(a)}$ means the WC of the DNA strand characterizing *a*.
3. An initial state *s*_*i*_ is encoded as 3′  $\bar{I_{1}}\;\bar{X_{0}\cdots X_{i}}$ 5′.
4. An acceptance state *s*_*j*_ is encoded as 3′  $\bar{X_{j+1}\cdots X_{m}}\;\bar{I_{2}}$ 5′.

The computational process of sticker automata can be summarized in the following three steps [56].

**Step 1 (data preprocessing).**

(1) Synthesize some DNA strands characterizing an automaton and its input strings.

(2) Put all the DNA strands into the test tube T, and anneal to make sure that the strands and their WC complements can be hybridized completely. The process of base pairing and the placement of ligase can form complete or partial double-stranded DNA molecules.

**Step 2 (computation).**

After Step 1, there are two possible cases. If the input string is accepted by the automaton, the tube T contains only the complete double-stranded DNA molecules, which begin with an initiator sequence and terminate at a terminator sequence. Otherwise, there are partial double-stranded or single-stranded DNA molecules in T. For the second case, some fragments of the single-stranded DNA molecules which characterize the input strings are paired successfully with some single-stranded DNA molecules which characterize transitions, whereas other fragments of the single-stranded DNA molecules which characterize the input strings cannot be paired with any single-stranded DNA molecules which characterize transitions. Therefore, ribozymes called Mung Bean are poured into the test tube T to degrade the single-stranded DNA fragment and retain the complete double-stranded DNA molecules.

**Step 3 (output of results).**

The DNA molecules with different lengths can be separated using the electrophoretic technique. If there exist a variety of lengths of DNA molecules, this indicates that there are some partial double-stranded DNA molecules in T before we add the ribozymes, and the input string cannot be accepted by the automaton. Otherwise, T contains only complete double-stranded DNA molecules before we add the ribozymes, and the input string can be accepted by the automaton.

#### 2.6.2. DNA Model Checking

On the basis of sticker automata, a DNA-computing-based LTL model checking method has been presented [55], which can be denoted as algorithm TL-MC-DNA(DNACODE(A), x), where DNACODE(A) and x are two inputs of the algorithm, where A is an FSA expressing a run of a system, DNACODE(A) is an encoding with a sticker automaton for characterizing A, x = DNACODE(A(*f*)) is an encoding with a sticker automaton for characterizing A(*f*), and A(*f*) is an FSA model of a formula *f*. The scope of *f* includes all the basic LTL formulas and some popular LTL formulas (*f* formula) [55]. The output of the algorithm is yes or no, representing the result of the model checking. The principle of this algorithm is illustrated by Figure 8.

### 2.7. The Four FSAs of the Formulas and Their DNA Model Checking

Given a temporal logic formula, an FSA model can be computed [43, 50, 52].  Figure 9 gives the four FSA models for the four specific formulas of temporal logic, respectively. Their corresponding relations are shown in Table 2, where *φ*_2_ and *φ*_3_ are the basic ITL formulas and *φ*_4_ is the basic PTL formula. In addition, *┐* is logical negation, $\overset{\text{-}\;\text{-}}{U}$ is logical duality of U, and $┐p\overset{\text{-}\;\text{-}}{U}┐q$ describes the property: for each path in M, there exists at least one state which does not satisfy *p*, before *q* is satisfied.

## 3. The DNA Model Checking Method

As mentioned in Section 2.6.2, if the encoding of one sticker automaton for an FSA of a system and the encoding of the other sticker automaton for an FSA of a formula are input into the algorithm TL-MC-DNA(DNACODE(A), DNACODE(A(f))) [55], the algorithm can compute and return the model checking results. This has been confirmed for the effectiveness of the algorithm TL-MC-DNA for the *f* formulas by simulated biological experiments [55]. This paper expands the range of the formula *f* and a series of new encodings of sticker automata, which will be explained in detail in Section 4. The DNA model checking for the four temporal logic formulas in Table 2 is performed by running the algorithm TL-MC-DNA(DNACODE(A), DNACODE(A(*f*′′))), where *f*′′ = {*φ*_1_, *φ*_2_, *φ*_3_, *φ*_4_} (*f*′′ formula). Section 4 will study the effectiveness of the new algorithm for the *f*′′ formulas by a number of simulated biological experiments. It should be noted that the algorithm TL-MC-DNA(DNACODE(A), DNACODE(A(*f*))) comes from previous research [55]. Due to space limitations, its pseudocode is not given in this paper.

### 3.1. The DNA Model Checking for the Basic CTL Formulas

There are eight basic CTL formulas. Due to the different semantics, the DNA model checking algorithms are different too.

#### 3.1.1. The DNA Model Checking for the Four Universal Formulas

Four basic CTL formulas, A*p*U*q*, AF*p*, AG*p*, and AX*p*, are called the universal formulas since their semantics are all involved in “all paths.” Comparing the CTL formula A*p*U*q* and the LTL formula *p*U*q*, it can be clearly seen that these two formulas have the same semantics. Therefore, the algorithm TL-MC-DNA(DNACODE(A), x) [55] can be employed to check the CTL formulas A*p*U*q*, AF*p*, AG*p*, and AX*p*. The detailed algorithm is formulated as shown in Algorithm 1.

#### 3.1.2. The DNA Model Checking for the Four Existence Formulas

The remaining four basic CTL formulas, E*p*U*q*, EF*p*, EG*p*, and EX*p*, are called the existence formulas since their semantics are all involved in “there exists at least one path.” Each of these four existence formulas is related to one of the universal formulas, which is summarized in Table 3.

Comparing $A┐p\overset{\text{-}\;\text{-}}{U}┐q$ and $\varphi_{1}=┐p\overset{\text{-}\;\text{-}}{U}┐q$, it can be observed that these two formulas have the same semantics. Thus, *┐φ*_1_ = E*p*U*q*. Therefore, the algorithm TL-MC-DNA(DNACODE(A), DNACODE(A(*f*′′ = *φ*_1_))) can be used to check the CTL formula E*p*U*q*. Similarly, the algorithm TL-MC-DNA(DNACODE(A), x) can be employed to check the CTL formulas EF*p*, EG*p*, and EX*p*. The detailed algorithm is formulated as shown in Algorithm 2. It should be noted that when a negative form of an atomic proposition occurs in the algorithm and is assigned as its argument, only one new atomic proposition is needed in the design of DNA encoding. No modification is needed on the algorithm, the FSA structure, or the encoding scheme of sticker automata.

#### 3.1.3. The DNA Model Checking for the Basic CTL Formulas

The principle of this algorithm is as follows. (1) If a basic CTL formula is a universal formula, Algorithm 1 will be called. (2) And if a basic CTL formula is an existence formula, Algorithm 2 will be called. In this way, model checking of the basic CTL formulas can be conducted. The algorithm is formulated as shown in Algorithm 3.

#### 3.1.4. Complexity Analysis

The time complexity of the algorithm TL-MC-DNA is *O*(*m* + *n*) [55], where *m* means the number of nodes in an automaton and *n* means the number of edges in this automaton. Therefore, Algorithm 1 needs to execute *O*(*m* + *n*) + *O*(3) = *O*(*m* + *n*) times operations. Similarly, Algorithm 2 needs to execute *O*(*m* + *n*) + *O*(3) = *O*(*m* + *n*) times operations. Algorithm 3 calls Algorithm 1 or Algorithm 2, so that the complexity of Algorithm 3 is *O*(*m* + *n*). In comparison, the model checking of the basic CTL formulas based on classical computing has a square complexity.

Regarding the efficiency of the algorithm in the classical model checking based on electronic computing, a computational process will advance sequentially. In the DNA model checking, the process is different. A large number of molecules execute computations at the same time, in a parallel manner. Although/since the massive computational units (i.e., molecules) are involved in computation, the efficiency of the algorithm is improved. In contrast, the classical model checking requires fewer computational units but more computational steps. In short, the DNA computing is better in the time at the cost of space, compared with the classical computing. Thus, the two kinds of computing approaches are complementary.

### 3.2. The DNA Model Checking for the Basic ITL Formulas

#### 3.2.1. The DNA Model Checking for the Basic ITL Formulas

There are two basic ITL formulas. The basic ITL formula (*p*_1_U*q*_1_); (*p*_2_U*q*_2_) can perform DNA model checking by calling the algorithm TL-MC-DNA(DNACODE(A), DNACODE(A(*f*′′ = *φ*_2_))). The basic ITL formula (*p*U*q*)^*∗*^ can perform DNA model checking by calling the algorithm TL-MC-DNA(DNACODE(A), DNACODE(A(*f*′′ = *φ*_3_))). The algorithm is formulated as shown in Algorithm 4.

#### 3.2.2. Complexity Analysis

Algorithm 4 calls the algorithm TL-MC-DNA, which has a complexity of *O*(*m* + *n*) [55]. Therefore, the complexity of Algorithm 4 is *O*(*m* + *n*). In comparison, the model checking of the basic ITL formulas based on classical computing has an exponential complexity.

### 3.3. The DNA Model Checking for the Basic PTL Formula

#### 3.3.1. The DNA Model Checking for the Basic PTL Formula

The DNA model checking for the basic PTL formula ((*p*_1_U*q*_1_), (*p*_2_U*q*_2_)) prj (*p*_3_∧X*q*_3_) can be performed by calling the algorithm TL-MC-DNA(DNACODE(A), DNACODE(A(*f*′′ = *φ*_4_))). The algorithm is formulated as shown in Algorithm 5.

#### 3.3.2. Complexity Analysis

Algorithm 5 calls the algorithm TL-MC-DNA which has a complexity of *O*(*m* + *n*) [55]. Thus, the complexity of Algorithm 5 is *O*(*m* + *n*). In comparison, the model checking of the basic PTL formula based on classical computing has an exponential complexity.

## 4. Simulated Experiments

The core implement component of our new approaches is TL-MC-DNA algorithm which is called by all the new methods. We have implemented this algorithm on the general model of sticker automata, with a simulation platform called NUPACK [57]. It has been confirmed that, (1) for the nine FSAs of the nine specific temporal logic formulas, the algorithm TL-MC-DNA can be realized effectively in molecular biology; (2) for the above FSAs, one can design their appropriate encoding of sticker automata, so that the accuracy rate of base pairing reaches more than 99% [55]. For the four FSAs of the formulas presented in Section 2.7, it is important to implement the TL-MC-DNA algorithm effectively in molecular biology. In particular, the biological effectiveness of the algorithms from 1 to 5 is dependent on this. Therefore, the same experimental platform and experimental means with the ones in [55] are employed to carry out the molecular biological simulated experiments.

The design of the DNA encoding is in relation to the success of the experiment. In order to ensure the specificity of hybridization, an encoding sequence must satisfy some physical constraints and thermodynamic constraints [58]. In this paper, the thermodynamic constraints, including the thermal denaturizing temperature, and the free energy are studied only because the problem is limited by the physical constraints [55]. NUPACK can be employed to design the DNA encodings for sticker automata, and this tool can simulate the hybridization phenomena which originate from the running of the TL-MC-DNA algorithm. This experimental way has been proved to be scientific in [55].

**Figure 7:**
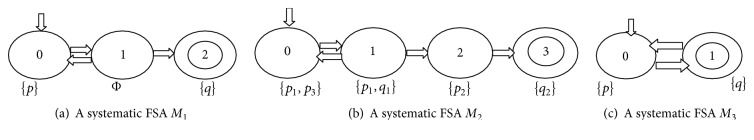
Some examples on LFSA: the systematic models of the experiments in this paper.

**Figure 8:**
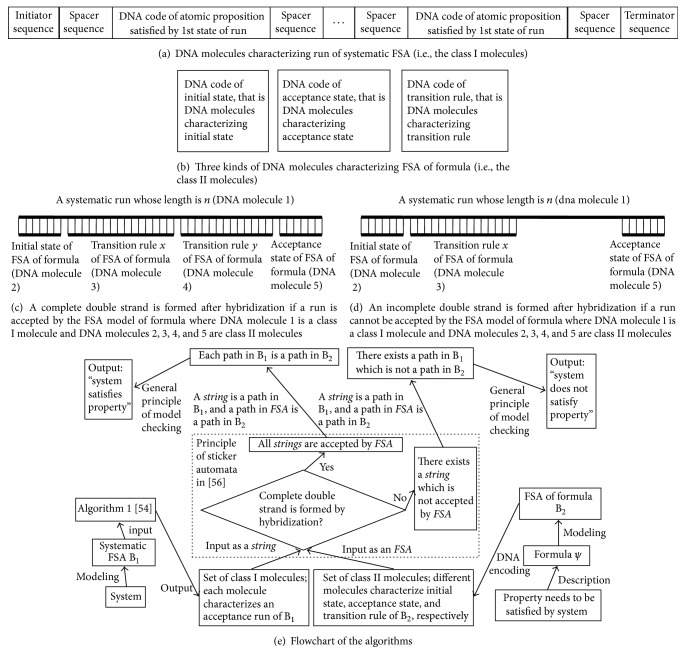
Principle of the model checking algorithms based on DNA computing [55].

**Figure 9:**
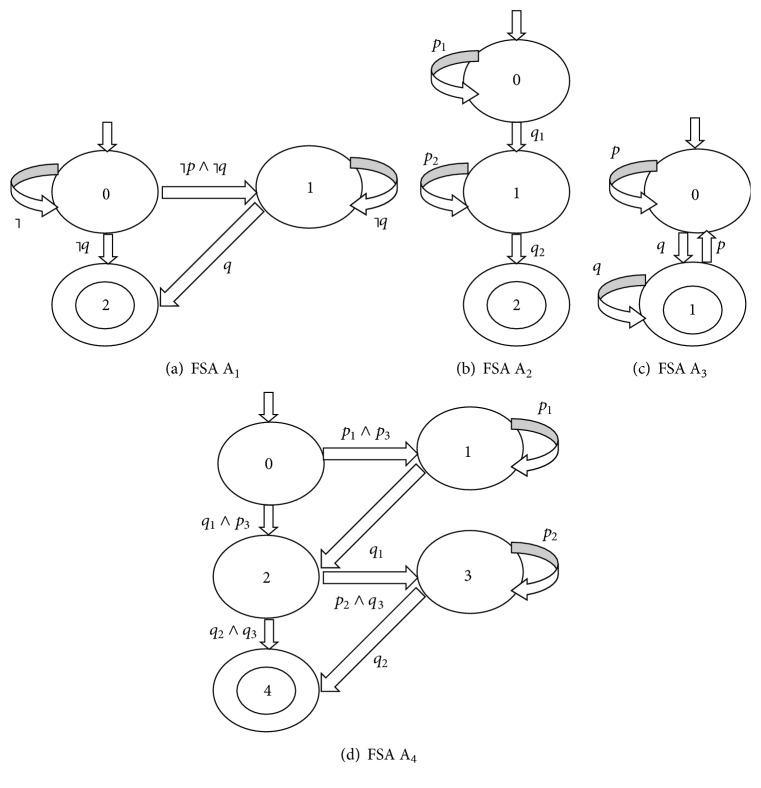
The four FSA models of the four formulas.

*Experimental Procedure*. (1) According to Figures 7 and 9, one can design the encoding of the sticker automata for systematic FSAs shown in each subgraph, as well as the encoding of the sticker automata for FSAs of formulas shown in each subgraph, respectively; (2) for these FSAs mentioned above, one can simulate the process of hybridization between some single-stranded DNA molecules; (3) according to the five algorithms proposed in this paper, one can get the results of model checking of various temporal logic formulas, by reading the results of hybridization.

*Experimental Objective*. The objective is to test the correctness, effectiveness, and biological reliability of the new algorithms.

### 4.1. Simulated Experiments for *φ*_1_

#### 4.1.1. Encoding Designs

**Figure 10:**
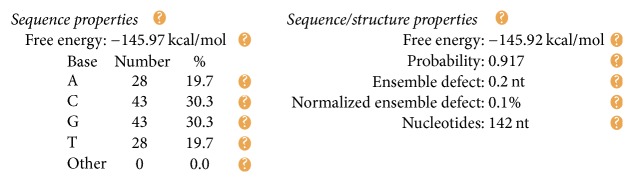
Checking the formula *φ*_1_: the structural properties of encoding sequence.

**Figure 11:**
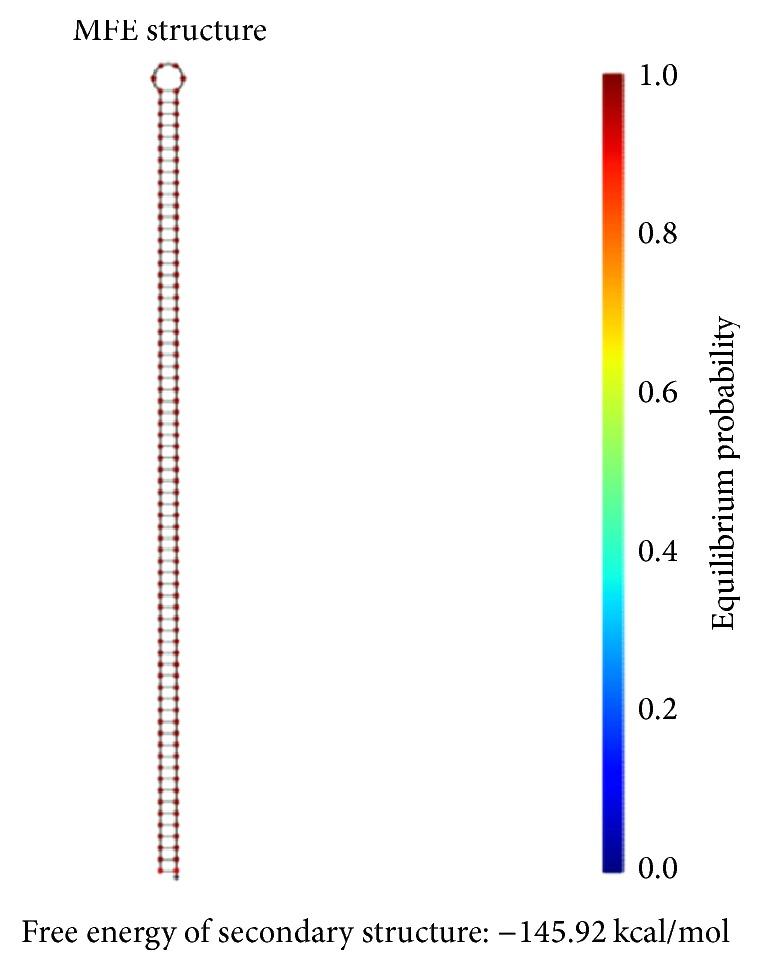
Thermodynamic analysis for *φ*_1_: minimum free energy structure.

**Figure 12:**
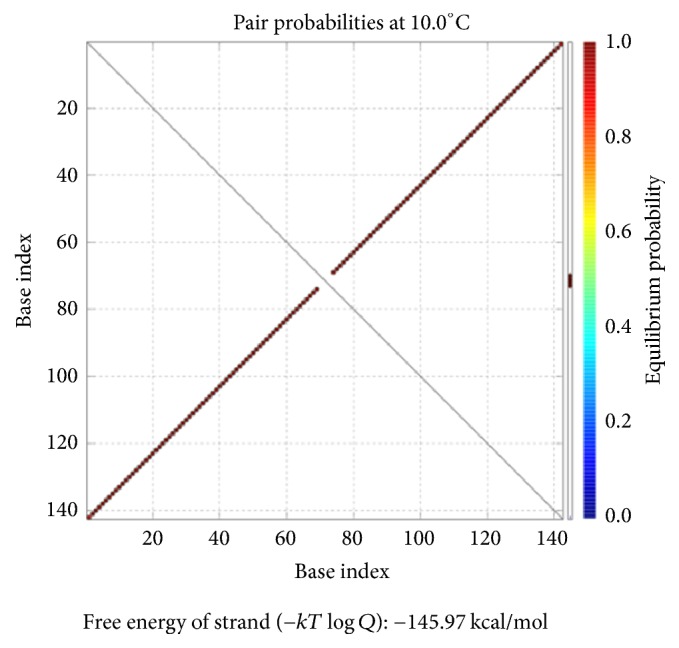
Checking for *φ*_1_: pairing probability in equilibrium.

The DNA encoding via NUPACK is designed, as illustrated in Table 4. Figures 10, 11, and 12 show the thermodynamic analysis of the encoding sequence at 10°C. As shown in Figure 10, the Normalized Ensemble Defect (NED) means the incorrect matching ratio of the nucleotides when a biochemical reaction is in equilibrium. 0% implies an optimal design, whereas 100% implies the worst design. The NED of our coding sequence is 0.1%.

The principle of the minimum free energy points out that the free energy is minimized when a biochemical reaction is in equilibrium. As shown in Figure 11, the color of the match between two kinds of molecules is dark red. The probability of the following event almost reaches 100%: the double-stranded molecule is completely matched. We find this fact by comparing color changes of the vertical bar that indicate the balance probability. Thus, its free energy is approximately equal to the minimum free energy.

As shown in Figure 12, the position of the red line indicates that all bases in the two single strands are completely complementary to each other, and the color of the red line indicates that the probability of all the pairs is approximately equal to 1. As analyzed above, our DNA sequence satisfies the minimum free energy constraint and the DNA molecules that participate in the reaction have a basically consistent temperature of solution chain. Therefore, the experimental results obtained from this encoding are reliable and effective in biology.

In fact, Table 4 indicates the encoding rules for the input strings, as shown in Table 5. According to Table 5 and the principle of encoding of sticker automata, we can deduce the encoding of the sticker automaton characterizing *φ*_1_, as shown in Table 6.

#### 4.1.2. Simulated Experiments

With the DNA code given in Section 4.1.1 at hand, we can conduct our simulated experiments. It should be noted that, in Section 4.1.2, all the encoding of the DNA molecules is written from left to right with a 5′-3′ direction, which is consistent with the way of writing in NUPACK.

We will check whether or not the systematic FSA *M*_1_ satisfies the formula *φ*_1_. According to the DNA codes given by Section 4.1.1, we can get all the paths which come from the systematic runs, as shown in Table 7, where *k* is a natural number. The transition rules shown in Table 6 clearly indicate that none of the atomic proposition excerpts for *s*, *u*, and *q* takes part in the transitions of states. Therefore, we do no need to consider whether or not the states satisfy the atomic propositions *p* and *r*.

First, we will check path 1. There are two possible runs in this path. Without loss of generality, we support that the atomic proposition sequence which is crossed by the run is *suq*.

All the molecules expressing the runs begin with GCCAGAA and end with GGCCGTC. Thus, we only need to consider *d* = TTGCAAGGCAGCGAATTGCAAGGCGCGGAATTGCAAGGCCCCGAATTGCAA. In short, we will observe whether or not hybridization occurs between the DNA molecules expressing transitions and the molecule *d*. For this experiment, the following six kinds of molecules are poured into a container with a volume of 10^−15^ L: *d*, *t*0*s*0, *t*0*u*1, *t*0*s*2, *t*1*s*1, and *t*1*q*2, for observing the hybridization.

The systematic run, which is expressed by the molecule *d*, crosses the three states. If hybridization occurs between the DNA molecules expressing transitions and the molecule *d*, there are not more than three kinds of molecules which are the WC of some segment of *d*, involved in the specific hybridization. For selecting three kinds of molecules from all the five kinds of WC molecules, one has ten choices. Thus, the following ten groups of subexperiments are performed, accordingly.

**Figure 13:**
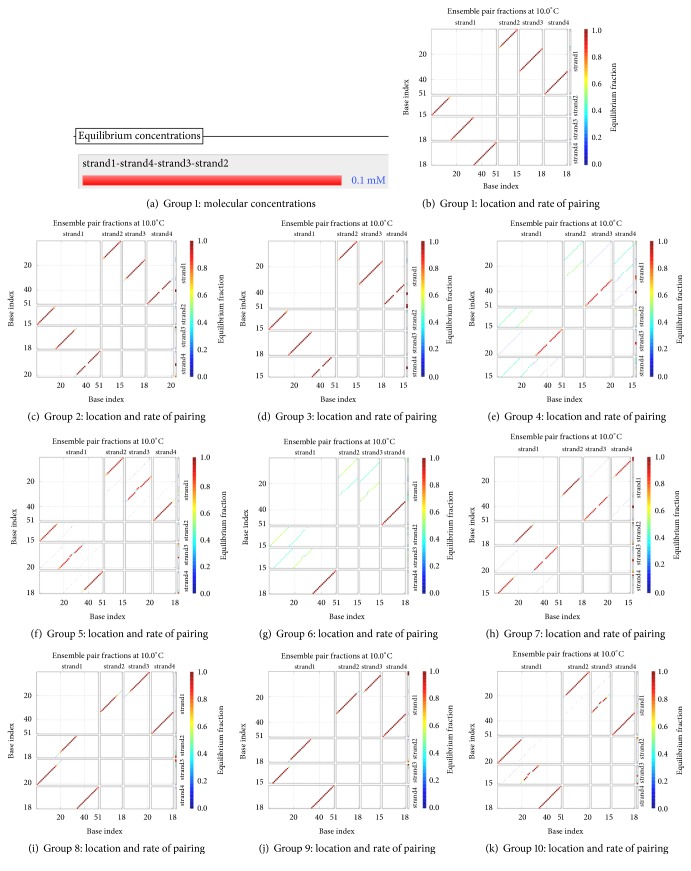
Checking for *φ*_1_: the groups of subexperimental results on base pairing and hybridization.

*(1) Group 1: t0s0, t0u1, t1q2, and d*. The concentrations of the four kinds of molecules are all 100 uM, and their molecular numbers are all 60000. With the temperature naturally dropped to 10°C, the hybridization reaction is observed. Figures 13(a) and 13(b) show the result of the hybridization, where strand1, strand2, strand3, and strand4 mean *d*, *t*0*s*0, *t*0*u*1, and *t*1*q*2, respectively.

In Figure 13(b), the coordinates of the location of the first red line from top to bottom indicate that the base sequence of the molecule *d* from the 1st to the 15th sites at 5′-3′ direction is paired with all of the fifteen bases of the molecule *t*0*s*0 at 3′-5′ direction. The coordinates of the location of the second red line from top to bottom indicate that the base sequence of the molecule *d* from the 16th to the 33rd sites at 5′-3′ direction is paired with all of the eighteen bases of the molecule *t*0*u*1 at 3′-5′ direction. The coordinates of the location of the third red line from top to bottom indicate that the base sequence of the molecule *d* from the 34th to the 51st sites at 5′-3′ direction is paired with all of the eighteen bases of the molecule *t*1*q*2 at 3′-5′ direction. The results suggest that the complete double-stranded DNA molecules are formed, and the hybridization among the four kinds of single-stranded DNA molecules is specific.

Comparing the color of the three red lines with the color change of the vertical bar on the right side of Figure 13(b), it can be clearly seen that the former colors are very close to the color at the top of the vertical bar. This phenomenon suggests that the probabilities of these base pairs are close to 100%. This is a higher degree of specificity.

As shown in Figure 13(a), the concentration of the molecule strand1-strand2-strand3-strand4 is 100 uM, and the concentrations of the molecules *t*0*s*0, *t*0*u*1, *t*1*q*2, and *d* are approximately equal to 0 after their hybridization. This indicates that all of the molecular reactants are involved in the specific hybridization, due to 100 uM/100 uM = 100%. Therefore, both the false negative rate and the false positive rate are approximate to 0. The true positive rate is approximately equal to 100%. In short, the results show that the four kinds of molecules are hybridized with strong specificity.

*(2) Group 2: t0s0, t0u1, t0s2, and d*. The concentrations of the four kinds of molecules are all 100 uM, and their molecular numbers are all 60000. As the temperature naturally drops to 10°C, the hybridization reaction is observed.  Figure 13(c) shows the result of the hybridization, where strand1, strand2, strand3, and strand4 mean *d*, *t*0*s*0, *t*0*u*1, and *t*0*s*2, respectively.

See Figure 13(c). There exists a red dot in the segment of strand1 of the vertical thin bar on the right side of strand4, indicating that some bases of strand1 are not paired with others. The results suggest that the four kinds of molecules in group 2 do not form complete double strands.

For all the other groups, all of the biochemical conditions and processes are similar to the groups above.

*(3) Group 3: t0s0, t0u1, t1s1, and d*.  Figure 13(d) shows the result. There exist some red dots in the segment of strand1 of the vertical thin bar on the right side of strand4, suggesting that the four kinds of molecules do not form complete double strands.

*(4) Group 4: t0s0, t0s2, t1s1, and d*.  Figure 13(e) shows the results. No red line is found at the 5′ end of strand1, indicating that the 5′ end of strand1 is not paired with any molecule. This suggests that the four kinds of molecules do not form complete double strands.

*(5) Group 5: t0s0, t0s2, t1q2, and d*. The results are shown in Figure 13(f). There exist some red dots in the segments of strand3 and strand4 of the vertical thin bar on the right side of strand4, suggesting that the four kinds of molecules do not form complete double strands.

*(6) Group 6: t0s0, t1s1, t1q2, and d*. As shown in Figure 13(g), no red line is found at the 5′ end of strand1, suggesting that the four kinds of molecules do not form complete double strands.

*(7) Group 7: t0u1, t0s2, t1s1, and d*. As shown in Figure 13(h), there are some red dots in the segments of strand1 of the vertical thin bar on the right side of strand4, suggesting that the four kinds of molecules do not form complete double strands.

*(8) Group 8: t0u1, t0s2, t1q2, and d*. As shown in Figure 13(i), there are some red dots in the segments of strand2 and strand3 of the vertical thin bar on the right side of strand4, suggesting that the four kinds of molecules do not form complete double strands.

*(9) Group 9: t0u1, t1s1, t1q2, and d*. As shown in Figure 13(j), there exist a red dot in the segments of strand1 of the vertical thin bar on the right side of the strand4, suggesting that the four kinds of molecules do not form the complete double strands.

*(10) Group 10: t0s2, t1s1, t1q2 and d*. As shown in Figure 13(k), there exist some red dots in the segments of strand2 and strand3 of the vertical thin bar on the right side of strand4, suggesting that the four kinds of molecules do not form complete double strands.

According to the ten groups of subexperiments mentioned above, we find that only group 1 (i.e., *t*0*s*0, *t*0*u*1, *t*1*q*2, and *d*) can form complete double strands by the hybridization reaction. That is to say, the systematic run *suq* satisfies the formula *φ*_1_, since the first state does not satisfy *q*, the second state satisfies none of *p* and *q*, and the third state satisfies *q*.

The above results are obtained when *k* = 1. It has been proved that a system satisfies the formula *p*U*q*, if and only if all the runs whose lengths are less than |*V* | *∗*2^|*V*|−1^+|*E*| satisfy *p*U*q*, where |*V*| and |*E*| mean the number of nodes and the number of edges in the systematic FSA, respectively [55]. Similarly, we can prove that this conclusion holds for *φ*_1_. *M*_1_ has three nodes and three edges. Thus, we need to check fifteen paths due to *k* = 3*∗*2^3−1^ + 3 = 15. With the same experimental way, we have checked the *k*th path, as shown in Table 8. *M*_1_ satisfies the formula *φ*_1_ since all paths (i.e., runs) satisfy this formula.

By calling the procedure for checking *φ*_1_, Algorithm 2 can get the model checking results on the formula E*p*U*q*. The model checking results on the eight basic CTL formulas are shown in Table 9. According to the experimental processes and results in Section 4.1, we can safely say that Algorithm 3, which can be employed to check the basic CTL formulas, has been effectively implemented in molecular biology.

### 4.2. Simulated Experiments for *φ*_2_ and *φ*_3_

#### 4.2.1. Encoding Designs

**Figure 14:**
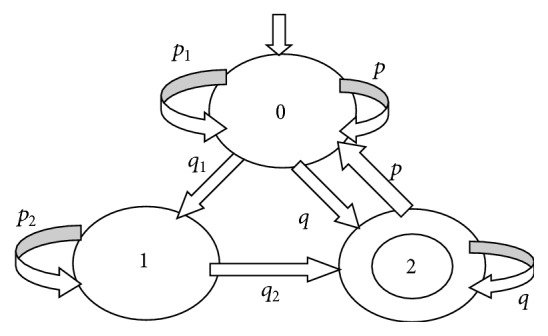
FSA of formula: merged graph A_5_ of A_2_ and A_3_.

**Figure 15:**
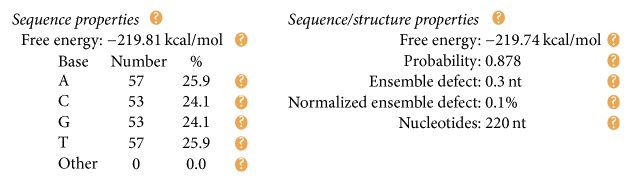
For *φ*_2_ and *φ*_3_: the structural properties of encoding sequence.

**Figure 16:**
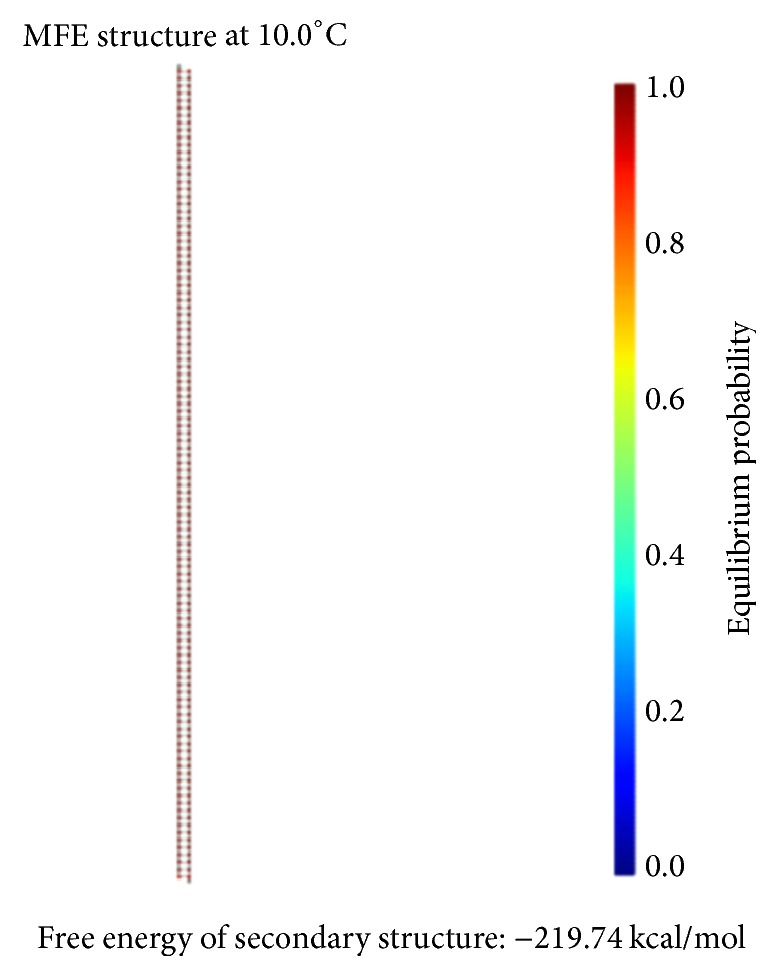
Thermodynamic analysis for *φ*_2_ and *φ*_3_: minimum free energy structure.

**Figure 17:**
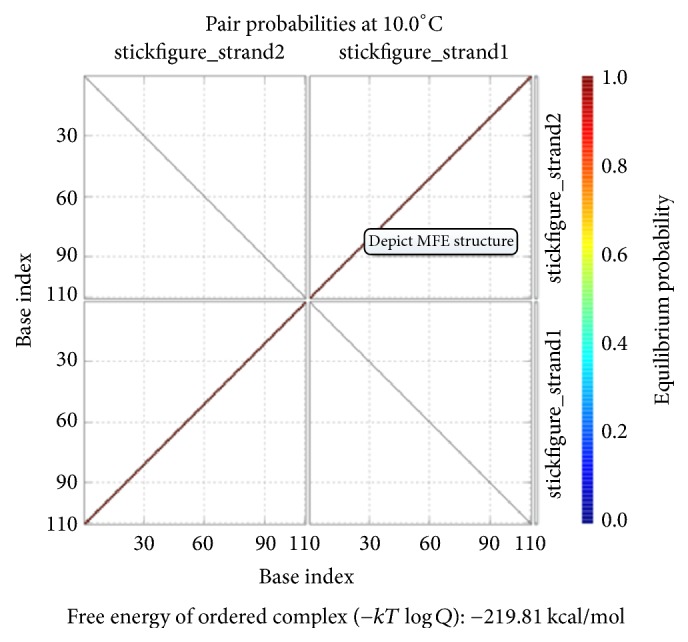
Checking for *φ*_2_ and *φ*_3_: pairing probability in equilibrium.

The formula *φ*_2_ and the formula *φ*_3_ need to be encoded with the same coding scheme since both formulas are ITL ones. Therefore, we combine the FSAs of these two formulas into one, as shown in Figure 14. Our design of a DNA encoding via NUPACK is shown in Table 10, while Figures 15, 16, and 17 show the thermodynamic analysis of the encoding sequence presented in Table 10 at 10°C. The NED of our coding sequence is 0.1%, which is illustrated in Figure 15. Its free energy is approximately equal to the minimum free energy, as shown in Figure 16. All bases in the two single strands are completely complementary to each other as shown in Figure 17, and the probabilities of all the pairs are approximately equal to 1. In Table 11, the encoding rules for the input strings are provided while Table 12 shows the encoding of the sticker automaton characterizing *φ*_2_ and *φ*_3_.

#### 4.2.2. Simulated Experiments

With the DNA code given in Section 4.2.1 at hand, we can conduct our simulated experiments. It should be noted that, in Section 4.2.2, all the encoding of the DNA molecules is written from left to right with a 5′-3′ direction, which is consistent with the way of writing using NUPACK.

*(1) Model Checking: Whether the Systematic FSA M*_2_ *Satisfies φ*_2_ *or Not*. With our DNA codes, all the paths of *M*_2_ are shown in Table 13, where *k* is a natural number. By observing the transition rules which are related to *φ*_2_ and shown in Table 12, it can be seen that none of the atomic proposition excerpts for *p*_1_, *q*_1_, *p*_2_, and *q*_2_ takes part in the transitions of states. Therefore, we do no need to consider whether or not the states satisfy other atomic propositions.

First, we will check path 1. There are two possible runs in this path. Without loss of generality, we suppose that the atomic proposition sequence which is crossed by the run is *p*_1_*q*_1_*p*_2_*q*_2_. We only need to deal with *d* = ATCGGAATGGATCGAATCGGAATGATACGAATCGGAATGGAACGAATCGGAATGTTCCGAATCGGA. In short, we will observe whether or not hybridization occurs between the DNA molecules expressing transitions and the molecule *d*. To this end, we pour the following five kinds of molecules into a container with a volume of 10^−15^ L: *d*, *t*0*p*_1_0, *t*0*q*_1_1, *t*1*p*_2_1, and *t*1*q*_2_2, for observing the hybridization.

**Figure 18:**
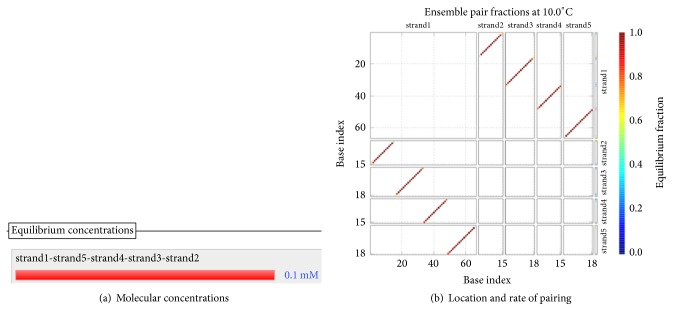
Checking for *φ*_2_: the experimental results on base pairing and hybridization.

The concentrations of the five kinds of molecules reach 100 uM, and their molecular numbers are all 60000. With the temperature naturally dropped to 10°C, the hybridization reaction is observed.  Figure 18 shows the result of the hybridization, where strand1, strand2, strand3, strand4, and strand5 mean *d*, t0*p*_1_0, *t*0*q*_1_1, *t*1*p*_2_1, and *t*1*q*_2_2, respectively.

In Figure 18(b), the coordinates of the location of the four red lines from top to bottom indicate that the complete double-stranded DNA molecules are formed by the hybridization among the five kinds of single-stranded DNA molecules. Comparing the color of the four red lines with the color change of the vertical bar on the right side of Figure 18(b), we can see clearly that the probabilities of these base pairs are close to 100%. This is a higher degree of specificity. As shown in Figure 18(a), the concentration of the molecules indicates that the true positive rate is approximately equal to 100%. Once again, it suggests that the five kinds of molecules are hybridized with strong specificity. Thus, the systematic run *p*_1_*q*_1_*p*_2_*q*_2_ satisfies the formula *φ*_2_.

The above results are gotten when *k* = 1. It has been proved that a system satisfies the formula *p*U*q*, if and only if all the runs whose lengths are less than |*V*|*∗*2^|*V*|−1^+|*E*| satisfy *p*U*q*, where |*V*| and |*E*| mean the number of nodes and the number of edges in the systematic FSA, respectively [55]. A system satisfies the formula *φ*_2_, if and only if all the runs whose lengths are less than (|*V*1|*∗*2^|*V*1|−1^ + |*E*1|)+(|*V*2|*∗*2^|*V*2|−1^ + |*E*2|) satisfy *p*U*q* since *φ*_2_ is composed of the two *p*U*q*-like formulas sequentially, where |*V*1| and |*E*1| mean the number of nodes and the number of edges in the prefix interval of the systematic FSA, respectively, and |*V*2| and |*E*2| mean the number of nodes and the number of edges in the suffix interval of the systematic FSA, respectively. For *M*_2_, |*V*1 | = 2, |*E*1 | = 2, |*V*2 | = 2, and |*E*2 | = 1. Thus, we need to check eleven paths due to 2*∗*2^2−1^ + 2 + 2*∗*2^2−1^ + 1 = 11, as shown in Table 14. *M*_2_ satisfies the formula *φ*_2_ since all paths (i.e., runs) satisfy this formula. By calling the procedure for checking *φ*_2_, Algorithm 4 can get the model checking results on this basic ITL formula.

*(2) Model Checking: Whether the Systematic FSA M*_3_ *Satisfies φ*_3_ *or Not*. According to the DNA codes given by Section 4.2.1, we can get all the paths of *M*_3_, as shown in Table 15, where *k* is a natural number. The transition rules, related to *φ*_3_ and shown in Table 12, show that none of the atomic proposition excerpts for *p* and *q* takes part in the transitions of states. Therefore, we do no need to consider whether or not the states satisfy other atomic propositions.

First, we will check path 1. The atomic proposition sequence which is crossed by the run is *pq*. We only need to deal with *d* = ATCGGAATGTATCGAATCGGAATGTGACGAATCGGA. In short, we will observe whether or not hybridization occurs between the DNA molecules expressing transitions and the molecule *d*. To this end, we pour the following five kinds of molecules into a container with a volume of 10^−15^ L: *d*, *t*0*p*0, *t*0*q*2, *t*2*p*0, and *t*2*q*2, for observing the hybridization.

**Figure 19:**
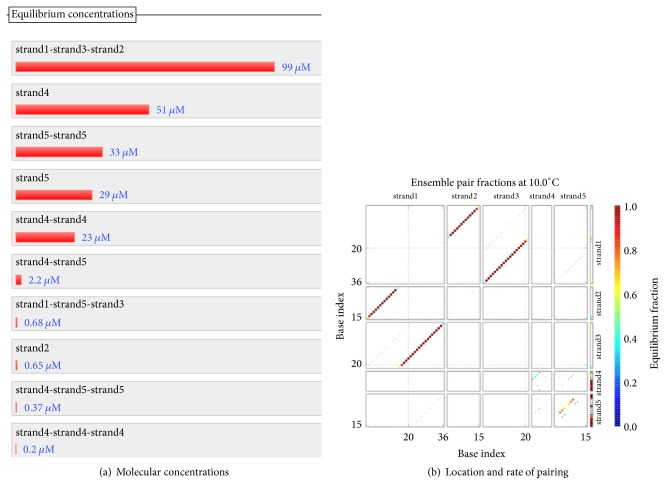
Checking for *φ*_3_: the experimental results on base pairing and hybridization.

The concentrations of the five kinds of molecules reach 100 uM, and their molecular numbers are all 60000. The hybridization reaction is observed as the temperature naturally drops to 10°C.  Figure 19 shows the result of the hybridization, where strand1, strand2, strand3, strand4, and strand5 mean *d*, *t*0*p*0, *t*0*q*2, *t*2*p*0, and *t*2*q*2, respectively.

As shown in Figure 19(b), the coordinates of the location of the two red lines from top to bottom indicate that the complete double-stranded DNA molecules are formed by the hybridization among *t*0*p*0, *t*0*q*2, and *d*. Comparing the color of the two red lines with the color change of the vertical bar on the right side of Figure 19(b), we can see clearly that the probabilities of these base pairs are close to 100%. This is a higher degree of specificity.

As shown in Figure 19(a), 99 uM/100 uM = 99% of the molecules *d* take part in the specific hybridization. Note that only the molecule strand1-strand3-strand2 is the product of the specific hybridization. Therefore, the true positive rate of the specific hybridization of *d* is approximately equal to 99%. Similarly, the false negative rate of *d* is equal to 0, and the false positive rate of *d* is 0.68 uM/100 uM = 0.68%. As for *t*0*p*0, its false negative rate is 0.65 uM/100 uM = 0.65%, its false positive rate is equal to 0, and its true positive rate is approximately equal to 99%. Similarly, the false negative rate of *t*0*q*2 is equal to 0, the false positive rate of *t*0*q*2 is 0.68 uM/100 uM = 0.68%, and the true positive rate of *t*0*q*2 is approximately equal to 99%. Once again, this suggests that the three kinds of molecules are hybridized with strong specificity. Thus, the systematic run *pq* satisfies the formula *φ*_3_.

Similarly, the above results are gotten when *k* = 1. It also has been proved that a system satisfies the formula *p*U*q*, if and only if all the runs whose lengths are less than |*V* | *∗*2^|*V*|−1^+|*E*| satisfy *p*U*q*, where |*V*| and |*E*| mean the number of nodes and the number of edges in the systematic FSA, respectively [55]. As for *φ*_3_, the same property holds, since *φ*_3_ is composed of the one *p*U*q*-like formula recursively. For *M*_3_, we need to check six paths due to 2*∗*2^2−1^ + 2 = 6, as shown in Table 16. *M*_3_ satisfies the formula *φ*_3_ since all paths (i.e., runs) satisfy this formula.

By calling the procedure for checking *φ*_3_, Algorithm 4 can get the model checking results on this basic ITL formula. According to the experimental processes and results in Section 4.2, we can safely say that Algorithm 4, which can be employed to check the basic ITL formulas, has been effectively implemented in molecular biology.

### 4.3. Simulated Experiments for *φ*_4_

#### 4.3.1. Encoding Designs

**Figure 20:**
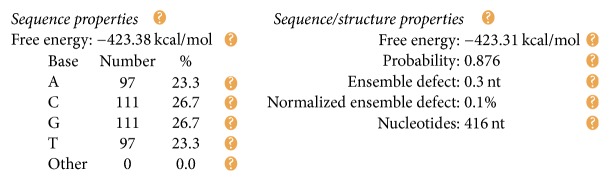
For *φ*_4_: the structural properties of encoding sequence.

**Figure 21:**
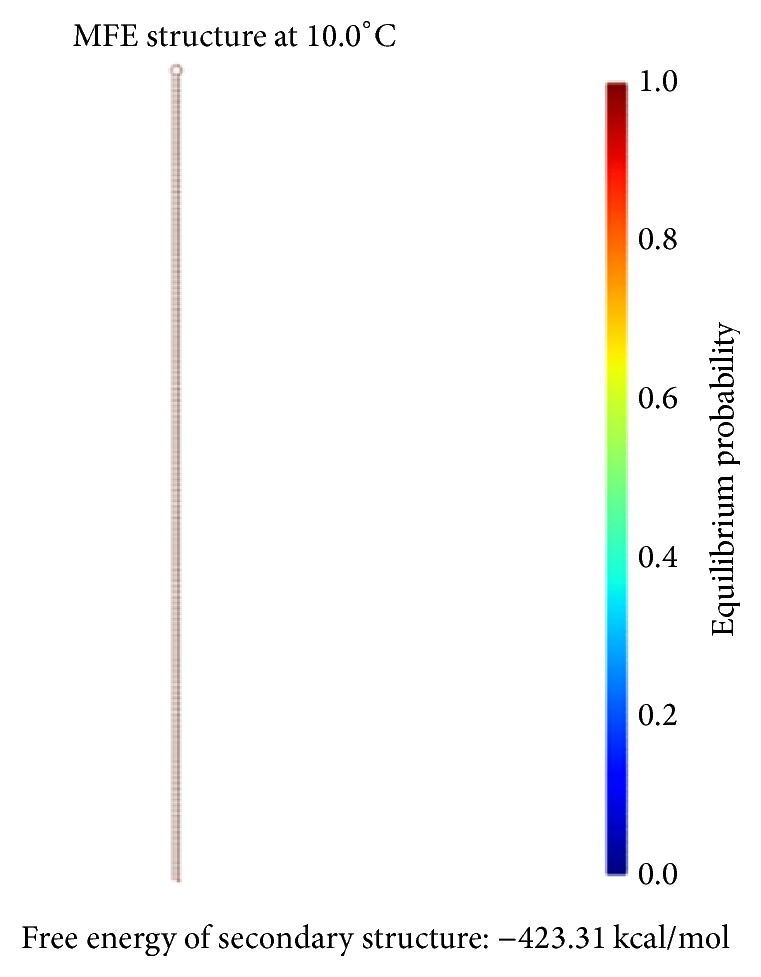
Thermodynamic analysis for *φ*_4_: minimum free energy structure.

**Figure 22:**
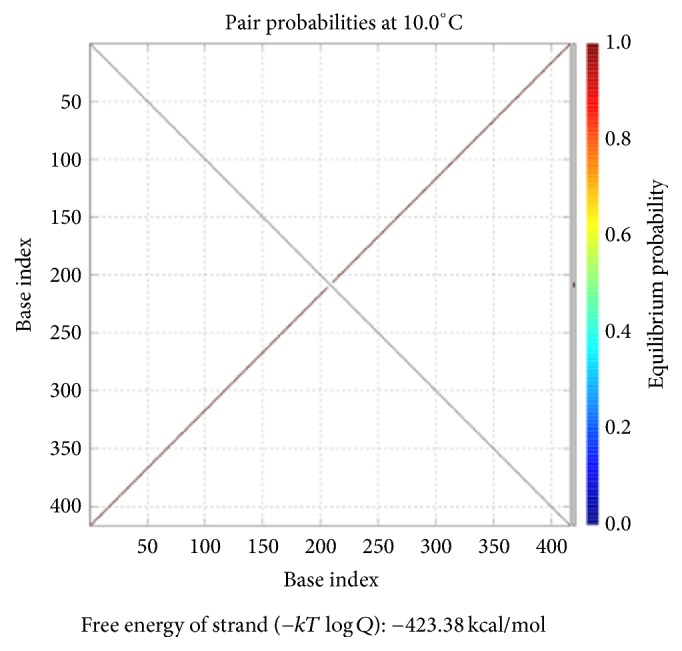
Checking for *φ*_4_: pairing probability in equilibrium.

We have designed a DNA encoding via NUPACK, as shown in Table 17. Figures 20, 21, and 22 show the thermodynamic analysis of the encoding sequence presented in Table 17 at 10°C. As shown in Figure 20, the NED of our coding sequence is 0.1%.  Figure 21 shows that its free energy is approximately equal to the minimum free energy and Figure 22 shows that all bases in the two single strands are completely complementary to each other, and the probabilities of all the pairs are approximately equal to 1.  Table 18 gives the encoding rules for the input strings. And Table 19 shows the encoding of the sticker automaton characterizing *φ*_4_.

#### 4.3.2. Simulated Experiments

With the DNA code given in Section 4.3.1 at hand, we can conduct our simulated experiments. It should be noted that, in Section 4.3.2, all the encoding of the DNA molecules is written from left to right with a 5′-3′ direction, which is consistent with the way of writing in NUPACK.

According to the DNA codes given by Section 4.3.1, we can get all the paths of *M*_2_, as shown in Table 20. The transition rules related to *φ*_4_ are shown in Table 19 and none of the atomic proposition excerpts for *p*_1_, *q*_1_, *p*_2_, *q*_2_, and *m*_1_ takes part in the transitions of states. Therefore, we do no need to consider whether or not the states satisfy other atomic propositions. First, we will check path 1. There are two possible runs in this path. Without loss of generality, we support that the atomic proposition sequence which is crossed by the run is *m*_1_*q*_1_*p*_2_*q*_2_. We only need to deal with *d* = CGCATCATGTGGTCTTTGCATGGACGTAGTGATCGGCGCATCATGTGGTCTTTGCATGGACGTAATCCTCGGCGCATCATGTGGTCTTTGCATGGACGTACAAATCGGCGCATCATGTGGTCTTTGCATGGACGTAGGGATCGGCGCATCATGTGGTCTT.

In short, we will observe whether or not hybridization occurs between the DNA molecules expressing transitions and the molecule *d*. For selecting four kinds of molecules from all the eight kinds of WC molecules, one has seventy choices. Thus, we need to execute the seventy groups of subexperiments. For example, we pour the following five kinds of molecules into a container with a volume of 10^−15^ L: *d*, *t*0*m*_1_1, *t*1*q*_1_2, *t*2*m*_3_3, and *t*3*q*_2_4, for observing the hybridization.

**Figure 23:**
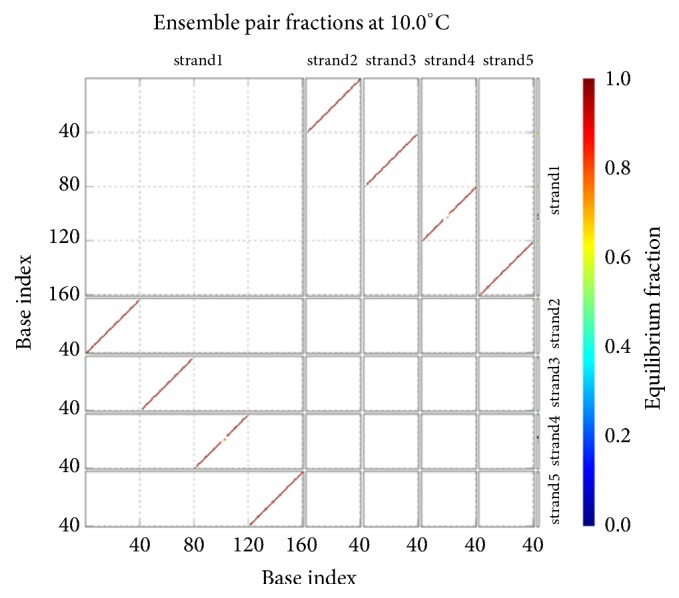
Checking for *φ*_4_: a group of subexperimental results on hybridization: location and rate of base pairing.

The concentrations of the five kinds of molecules reach 100 uM, and their molecular numbers are all 60000. With the temperature naturally dropped to 10°C, the hybridization reaction is observed.  Figure 23 shows the result of the hybridization, where strand1, strand2, strand3, strand4, and strand5 mean *d*, *t*0*m*_1_1, *t*1*q*_1_2, *t*2*m*_3_3, and *t*3*q*_2_4, respectively.

As shown in the graph, the bases in the middle of strand4 are not paired with strand1, indicating that the complete double strands are not formed. As for any other group of subexperiments, the complete double strands are not formed. Thus, the systematic run *m*_1_*q*_1_*p*_2_*q*_2_ does not satisfy the formula *φ*_4_. Similarly, none of the runs in path 1 satisfies this formula. Therefore, there exists a path which does not satisfy *φ*_4_. That is to say, *M*_2_ does not satisfy the formula *φ*_4_.

By calling the procedure for checking *φ*_4_, Algorithm 5 can get the model checking results on this basic PTL formula. According to the experimental processes and results in Section 4.3, we can safely say that Algorithm 5, which can be employed to check the basic PTL formulas, has been effectively implemented in molecular biology.

### 4.4. The Effect of Reaction Temperature on the Above Experimental Results

**Figure 24:**
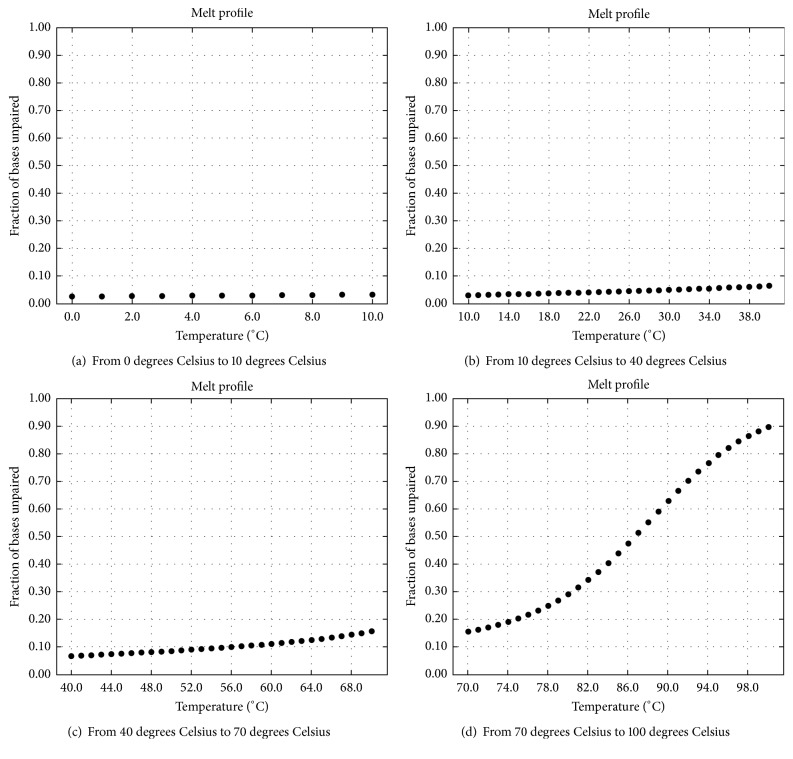
Effect of reaction temperature on hybridization for *φ*_1_: forming complete double strands (rate of unpaired bases).

**Figure 25:**
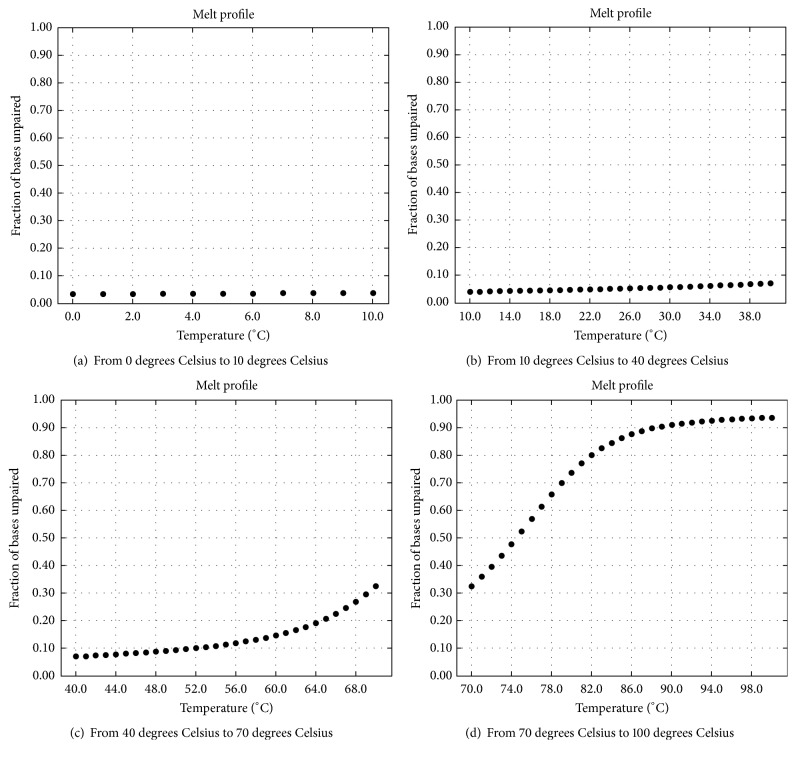
Effect of reaction temperature on hybridization for *φ*_2_: forming complete double strands (rate of unpaired bases).

**Figure 26:**
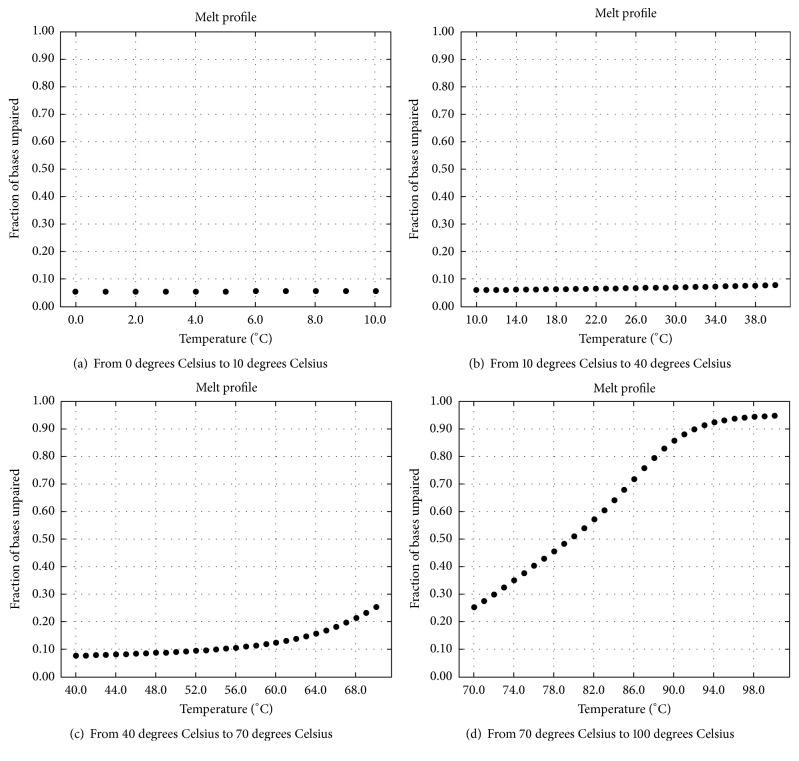
Effect of reaction temperature on specific hybridization for *φ*_3_: forming complete double strands (rate of unpaired bases).

As mentioned above, (1) the complete double strands shown in Figures 13(a) and 13(b) are formed in the process of checking *φ*_1_; (2) the complete double strands shown in Figure 18 are formed in the process of checking *φ*_2_; (3) the complete double strands shown in Figure 19 are formed in the process of checking *φ*_3_. For these complete double strands which come from the hybridization, the relationships between the rates of unpaired bases and the reaction temperatures are illustrated in Figures 24, 25, and 26.

The temperatures are illustrated in Figures 24, 25, and 26. As shown in these graphs, the lower the reaction temperature, the higher the ratio of base pairing. This result suggests that the cooling target temperature (i.e., reaction temperature) has an important influence on the experimental results, and 10°C is a suitable temperature to ensure the specificity of hybridization. In comparison, the initial temperature and the cooling speed are not crucial. As far as sticker automata are concerned, one can place directly a container with some molecular reactants at room temperature, in order to obtain his/her products of hybridization. This is the standard experimental way given in [56] for sticker automata.

### 4.5. The Simulated Experiments on Molecular Kinetics

**Figure 27:**
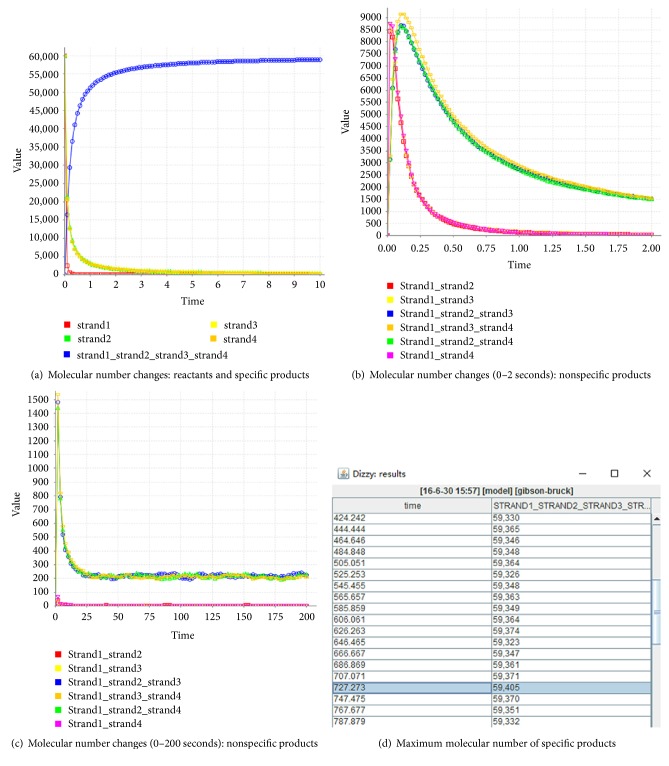
Simulated experiments in molecular kinetics: complete double strands formed for checking *φ*_1_.

**Figure 28:**
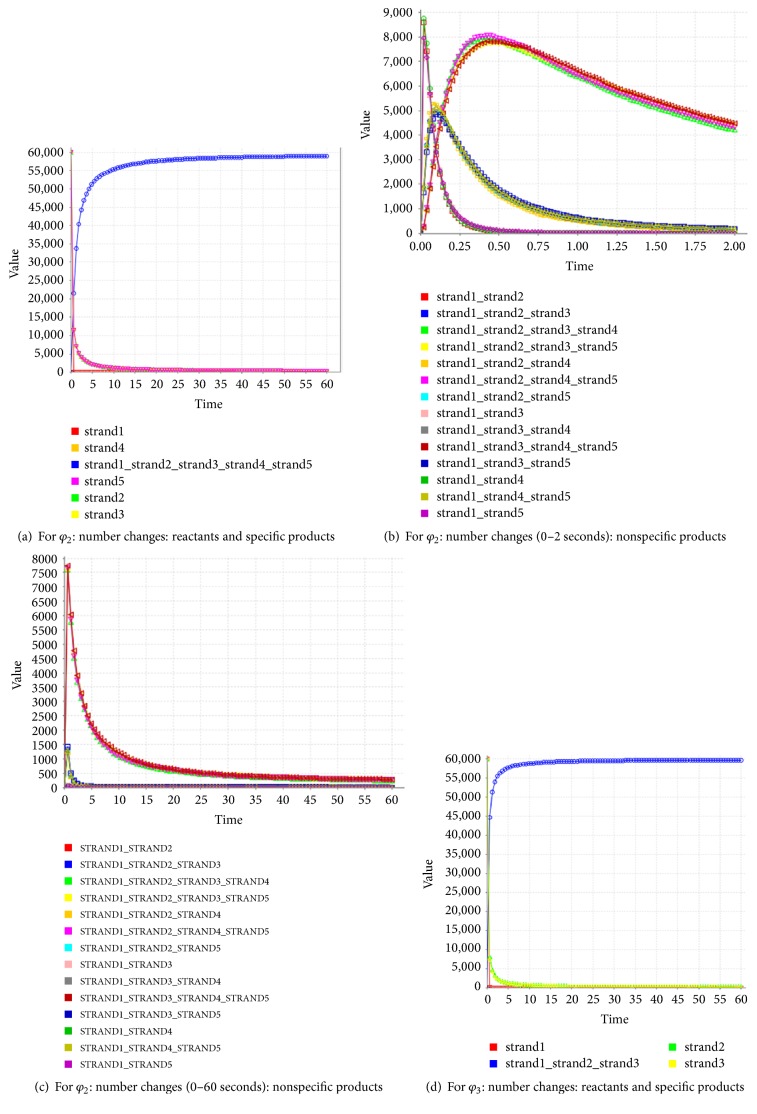
Experiments in molecular kinetics: complete double strands formed for checking *φ*_2_ and *φ*_3_.

Regarding the complete double strands shown in Figures 13(a), 13(b), 18, and 19, the base pairings are illustrated in the corresponding graphs. This is a result of the competitive hybridization among the different kinds of molecules. In order to better observe the process of molecular competition, we design a number of experiments. With the DIZZY tool for the DNA molecular kinetics [59], the famous chemical kinetics algorithm, called Gibson-Bruck [56], is applied to compute the dynamic changes of the numbers of the various kinds of molecules in the process of hybridization. The results are illustrated in Figures 27 and 28.

Figure 27 shows the variation of the numbers of the different kinds of molecules in the process of formation of the complete double strands shown in Figures 13(a) and 13(b). The blue line in Figure 27(a) shows a change in the number of the complete double strands. Throughout the process, the number of the complete double-stranded molecules increases exponentially with time elapse, and the number of the molecular reactants decreases exponentially with time elapse, approaching zero, as shown in Figure 27(a). Within 10 seconds, the blue line begins to approximate the upper bound of 60000 and it reaches the maximum value of 59405 (more than 99% of the upper bound) in 727 seconds (about 12 minutes), as shown in Figure 27(d).

The changes for the numbers of the nonspecific molecular products are illustrated in Figures 27(b) and 27(c). At first, they increase rapidly, suggesting that a large number of nonspecific products occur. In the end, they decrease exponentially, suggesting that the specific molecular products dominate the competition eventually. It can be explained that the specific molecular products have more advantages on the physical structure and thermodynamic properties than the nonspecific molecular products.

Figure 28 shows the variation of the numbers of the different kinds of molecules in the process of formation of the following two kinds of complete double strands: the one shown in Figure 18 and the one in Figure 19. These phenomena, rules, and causes in molecular kinetics are similar to the ones in Figure 27.

### 4.6. Some Comparisons among the New Method and the Related Ones

#### 4.6.1. Comparing the New Methods with Other Related DNA-Based Ones

**Figure 29:**
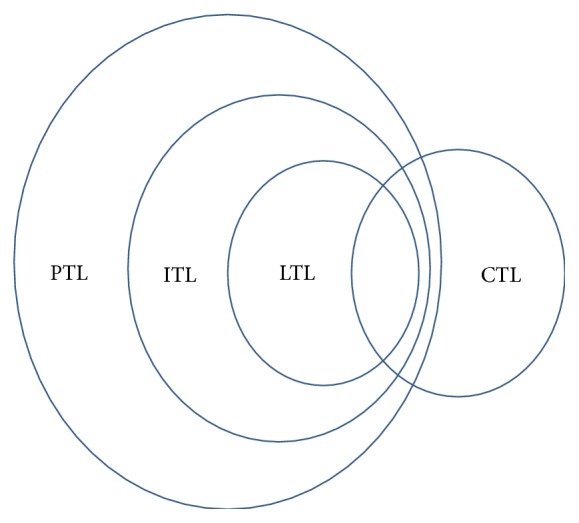
Comparison of power among the several logic types (comparison of action ranges among the new method and the existing ones).

Table 21 gives a comparison of power between the new method and the existing DNA-based ones. From this table, the following observations can be drawn:

- The DNA-computing-based approach for checking the basic CTL formula EF*p* [53] cannot deal with any other CTL formula (including the basic formula). In comparison, the new method can conduct model checking for all of the eight basic CTL formulas via DNA molecules. In addition, the method in [53] cannot deal with any ITL/PTL formulas, whereas the new method can deal with them.
- There are some previous DNA-computing-based approaches for checking all of the four basic LTL formulas and some popular LTL formulas [54, 55]. However, these methods cannot work on any of the CTL formula, ITL formula, and PTL formula. In comparison, the new method can conduct model checking for all of the basic formulas of the above three temporal logic types. In particular, the relationship of the expressive abilities of these three temporal logic types is shown in Figure 29.

In summary, our new method extends the range of the DNA model checking, and some stronger temporal properties can be checked. In addition, the new method does not simply call the algorithm TL-MC-DNA in [55]. There are some key differences between the two methods.

- First, the new approach has employed some formal technique based on the semantic equivalent transformations before calling the algorithm TL-MC-DNA.
- Second, the new approach extends the scope of input parameters of the algorithm TL-MC-DNA.
- Third, we have designed a number of the new DNA encoding schemes which are more effective and used together with the new algorithms.
- Fourth, the targeted problems are different. The algorithm TL-MC-DNA is used for the basic LTL model checking, whereas the new approach is used for the basic CTL model checking, the basic ITL model checking, and the basic PTL model checking. In other words, the latter problems are reduced to the former solved problem, using a series of logical ways and molecular biological ones. This is the research scheme in this paper.

In addition, Sections 4.1, 4.2, and 4.3 have confirmed that the simulated biochemical experiments can ensure the correctness and effectiveness for DNA model checking. In comparison, the simulated experiments on molecular kinetics in Section 4.5 further demonstrate that the DNA model checking can be biochemically implemented in acceptable time.

#### 4.6.2. Comparing the New Method to the Classical Model Checking Algorithms

As shown in Section 3.1.4, the model checking method using DNA molecules is different from the model checking algorithms based on electronic computing devices, in terms of the computing mechanism due to the different computing carriers. As a result, the new method and the classical ones in [43] are complementary.

#### 4.6.3. Additional Discussions

In [60], Professor Lamport talked about the problems that he knows with liveness. One problem is that “more than 90% (probably more than 95%) of the errors in real systems are violations of safety properties.” The CTL formula AF*p* is usually employed to describe a safety property in practical model checking. Our new method can check AF*p* via DNA molecules, as illustrated in Algorithm 1. Therefore, the core of CTL in this paper is useful in practice of computing.

Previous research has demonstrated that the DNA model checking technique using sticker automata can be implemented in molecular biology [55], if the number of the nodes of FSA of a logical formula is not greater than 7 and the number of the edges of FSA of the logical formula is not greater than 42. It is obvious that the new method aims to deal with CTL/ITL/PTL formulas using sticker automata. Thus, our molecular biology technique mentioned above indicates that not only all of the basic formulas but also some popular CTL/ITL/PTL formulas in practice can be dealt with, by extending the new method, which is similar to the case of LTL in [55]. In addition, the methods based on sticker automata can deal with the complete decidable sets of temporal logic using some extended DNA encoding, in theoretical computing [55]. The details of these extensions are omitted from the paper due to the scope of this paper and the limitation of space.

## 5. Conclusions

Early studies on DNA computing focused on nonautonomous models and algorithms. The DNA computing techniques have been optimized with self-assembly in recent years. This paper has presented a novel DNA computing method using sticker automata for model checking temporal logic formulas. Particularly, our newly developed algorithms are based on the self-assembly of sticker automata.

The state of the art of the universal DNA computer is encouraging [15, 30], and it is in a great need for new components to enhance the theoretical architecture of DNA computing, especially for the temporal logic model checking, which is a complex computational problem. The new algorithms have been implemented and model checking was conducted via DNA molecules for the basic formulas of CTL, ITL, and PTL. The model checking technique based on molecular computation has its intrinsic advantages of parallel computing, compared with the classical model checking methods. The new DNA computing approach based on sticker automata will develop a molecular solution and expand the previous DNA computational problem library.

There are several directions that can be further explored based on the new method described in this paper. One area is to apply the cellular model checking technique for genomic research. For example, it can be used to study DNA repairing and mismatching during cell division, which was believed to be associated with cancer occurrence [56]. In order to improve the ability of discovery and repair of abnormal genes not only at the structural level but also at the functional level, it is necessary to study the temporal and spatial expression of genes. Some previous research has employed the cellular computation technique to provide an autonomous intelligent method for the molecular diagnosis and treatment of some human diseases which are caused by genetic mutation [56]. However, the new method can deal with more temporal relationships. Therefore, one of the future works of our study is to incorporate our new DNA computing approach for model checking temporal logic into the artificial controlled gene repair techniques. This will develop a molecular means for the early detection, diagnosis, and treatment of cancer. It will impact the prognosis and the survival rate of cancer patients.

A specific future application of our method is to study the gastric cardiac cancer for the stage of gastric inflammation and precancerous lesions, which showed some abnormal behavioral changes in genes with temporal characteristics. To give an example, previous study discovered a susceptibility gene locus of gastric cardiac cancer in the Han population of Northern China [61]. Future research will focus on how to embed the autonomous model checking method into living human cells. Such an approach can be used to develop a molecular robot technique to repair susceptibility loci or DNA mismatches in the gastric cancer cells or the normal cells. To be a little more specific, the basic CTL formulas can be applied to describe the branch temporal relationships among dynamic changes in genes, the basic ITL formulas for the simple interval relationships among dynamic changes in genes, and the basic PTL formula for the general relationships between intervals and their effects of dynamic changes in genes. These basic temporal logical formulas are sufficient to describe the dynamic changes of genes and no other formulas are needed.

## Figures and Tables

**Algorithm 1:**
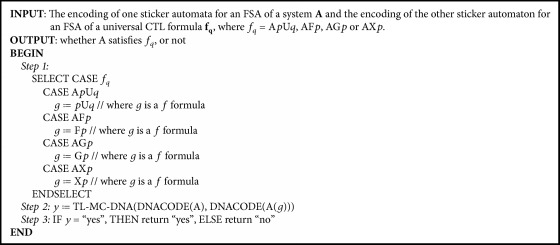
CTLQ-MC-DNA(DNACODE(A), DNACODE(A(*f*_*q*_))), the DNA model checking algorithm for the universal CTL formulas.

**Algorithm 2:**
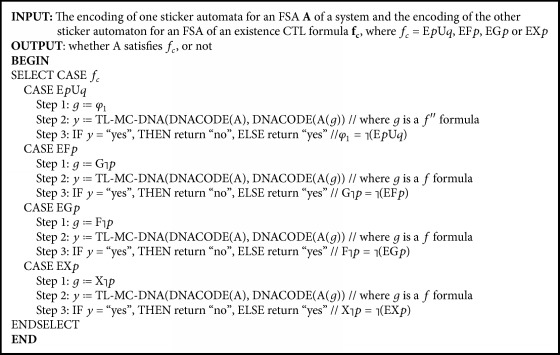
CTLC-MC-DNA(DNACODE(A), DNACODE(A(*f*_*c*_))), the DNA model checking algorithm for the existence CTL formulas.

**Algorithm 3:**
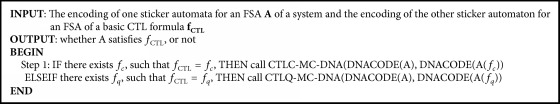
CTL-MC-DNA(DNACODE(A), DNACODE(A(*f*_CTL_))), the DNA model checking algorithm for the basic CTL formulas.

**Algorithm 4:**
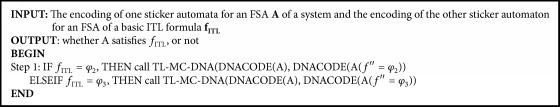
ITL-MC-DNA(DNACODE(A), DNACODE(A(*f*_ITL_))), the DNA model checking algorithm for the basic ITL formulas.

**Algorithm 5:**
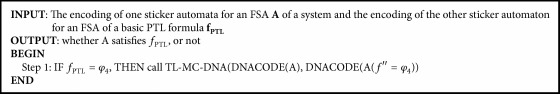
PTL-MC-DNA(DNACODE(A), DNACODE(A(*f*_PTL_))), the DNA model checking algorithm for the basic PTL formulas.

**Table 1:** State of the art of DNA model checking and its open problems.

Temporal logic	State of the art (the formulas which have been checked)	The problems to be solved (the formulas which cannot be checked yet)
LTL	All the four basic formulas	General formulas and cellular model checking
CTL	The basic formula EF*p*	Another seven basic formulas (will be studied in this paper)
ITL	Nothing reported	All the two basic formulas (will be studied in this paper)
PTL	Nothing reported	All the one formula (will be studied in this paper)
DC	It is infeasible due to the limitation of the current biochemical experimental technique	

**Table 2:** Relationships: the four formulas of temporal logic and their FSA models, where $\overset{\text{-}\;\text{-}}{U}$ is the logical duality of U.

The formula	$\varphi_{1}=┐p\overset{\text{-}\;\text{-}}{U}┐q$	*φ*_2_ = (*p*_1_U*q*_1_); (*p*_2_U*q*_2_)	*φ*_3_ = (*p*U*q*)^*∗*^	*φ*_4_ = ((*p*_1_U*q*_1_), (*p*_2_U*q*_2_)) prj (*p*_3_∧X*q*_3_)
FSA of formula	A_1_	A_2_	A_3_	A_4_

**Table 3:** The relationships between the existence and the universal CTL formulas.

Existence formulas	Universal formulas	Relationships
E*p*U*q*	A*p*U*q*	$┐EpUq=A┐p\overset{\text{-}\;\text{-}}{U}┐q$
		$EpUq=┐A┐p\overset{\text{-}\;\text{-}}{U}┐q$
EG*p*	AF*p*	*┐*EG*p* = AF*┐p*
		EG*p* = *┐*AF*┐p*
EF*p*	AG*p*	*┐*EF*p* = AG*┐p*
		EF*p* = *┐*AG*┐p*
EX*p*	AX*p*	*┐*EX*p* = AX*┐p*
		EX*p* = *┐*AX*┐p*

**Table 4:** Checking for *φ*_1_: the designed encoding sequence, where WC means Watson-Crick complementary strand of code.

Code	5′ GCCAGAATTGCAAGGCAGCGAATTGCAAGGCGCGGAATTGCAAGGCCCCGAATTGCAAGGCCGTCCGACGC 3′
WC	3′ CGGTCTTAACGTTCCGTCGCTTAACGTTCCGCGCCTTAACGTTCCGGGGCTTAACGTTCCGGCAGGCTGCG 5′

**Table 5:** Checking for *φ*_1_: the encoding rules of input strings characterizing runs, encoding by the way of sticker automata.

Object of code	DNA code
Initiator sequence	*I*_1_ = 5′ GCCA 3′
Spacer sequence	*X*_0_ = 5′ GAA 3′, *X*_1_ = 5′ TTG 3′, *X*_2_ = 5′ CAA 3′, *X*_3_ = 5′ GGC 3′
Terminator sequence	*I*_2_ = 5′ CGTC 3′
Atomic proposition	*p* = 5′ CGA 3′, *q* = 5′ CCC 3′, *r* = ¬*p* = 5′ CGC 3′, *s* = ¬*q* = 5′ AGC 3′, *u* = *r*∧*s* = 5′ GCG 3′

**Table 6:** Checking for *φ*_1_: the encoding of FSA A_1_ of formula, encoding by the way of sticker automata, where sto() means WC.

Object of code	Abbreviated transition rule	DNA code
Initial state *s*_0_	None	3′ sto(*I*_1_ *X*_0_) 5′ = 3′ CGGTCTT 5′
Acceptance state *s*_2_	None	3′ sto(*X*_3_ *I*_2_) 5′ = 3′ CCGGCAG 5′
Transition rule *t*(*s*_0_, *s*) = *s*_0_	*t*0*s*0	3′ sto(*X*_1_*X*_2_*X*_3_ *s* *X*_0_) 5′ = 3′ AACGTTCCGTCGCTT 5′
Transition rule *t*(*s*_0_, *u*) = *s*_1_	*t*0*u*1	3′ sto(*X*_1_*X*_2_*X*_3_ *u* *X*_0_*X*_1_) 5′ = 3′ AACGTTCCGCGCCTTAAC 5′
Transition rule *t*(*s*_0_, *s*) = *s*_2_	*t*0*s*2	3′ sto(*X*_1_*X*_2_*X*_3_ *s* *X*_0_*X*_1_*X*_2_) 5′ = 3′ AACGTTCCGTCGCTTAACGTT 5′
Transition rule *t*(*s*_1_, *s*) = *s*_1_	*t*1*s*1	3′ sto(*X*_2_*X*_3_ *s* *X*_0_*X*_1_) 5′ = 3′ GTTCCGTCGCTTAAC 5′
Transition rule *t*(*s*_1_, *q*) = *s*_2_	*t*1*q*2	3′ sto(*X*_2_*X*_3_ *q* *X*_0_*X*_1_*X*_2_) 5′ = 3′ GTTCCGGGGCTTAACGTT 5′

**Table 7:** The runs of the system *M*_1_.

Path	DNA code of the path or sequence of nodes (atomic propositions) crossed by the path
Code of path 1	GCCA GAATTGCAAGGC AGC GAATTGCAAGGC AGC | GCG GAATTGCAAGGC CCC GAATTGCAAGGC CGTC
Sequence of nodes crossed by path 1	0,1, 2 (*s*, *s* | *u*, *q*)
Code of path *k*	GCCA GAATTGCAAGGC (AGC GAATTGCAAGGC AGC | GCG GAATTGCAAGGC)^k^ CCC GAATTGCAAGGC CGTC
Sequence of nodes crossed by path *k*	(0,1)^*k*^, 2

**Table 8:** The results: checking for *φ*_1_ in the different paths of *M*_1_ (whether or not the path satisfies *φ*_1_).

Formula	Path 1	Path *k*, where 15 > *k* > 1	Path 15	Does *M*_1_ satisfy *φ*_1_?
*φ*_1_	Yes	Yes	Yes	Yes

**Table 9:** The model checking results: *M*_1_ and the basic CTL formulas (whether or not the system *M*_1_ satisfies these formulas).

Formula	Result	The used algorithm and decision basis
A*p*U*q*	No	TL-MC-DNA determines that *M*_1_ does not satisfy *p*U*q*, and thus Algorithm 1 determines that *M*_1_ does not satisfy A*p*U*q*
AF*p*	Yes	TL-MC-DNA determines that *M*_1_ satisfies F*p*, and thus Algorithm 1 determines that *M*_1_ satisfies AF*p*
AG*p*	No	TL-MC-DNA determines that *M*_1_ does not satisfy G*p*, and thus Algorithm 1 determines that *M*_1_ does not satisfy AG*p*
AX*p*	No	TL-MC-DNA determines that *M*_1_ does not satisfy X*p*, and thus Algorithm 1 determines that *M*_1_ does not satisfy AX*p*
E*p*U*q*	No	Extended TL-MC-DNA determines that *M*_1_ satisfies *φ*_1_, and thus Algorithm 2 determines that *M*_1_ does not satisfy E*p*U*q*
EF*p*	Yes	TL-MC-DNA determines that *M*_1_ does not satisfy G¬*p*, and thus Algorithm 2 determines that *M*_1_ satisfies EF*p*
EG*p*	No	TL-MC-DNA determines that *M*_1_ satisfies F¬*p*, and thus Algorithm 2 determines that *M*_1_ does not satisfy EG*p*
EX*p*	No	TL-MC-DNA determines that *M*_1_ satisfies X¬*p*, and thus Algorithm 2 determines that *M*_1_ does not satisfy EX*p*

**Table 10:** Checking for *φ*_2_ and *φ*_3_: the designed encoding sequence.

Code	5′ CGCTCGAATCGGAATGGATCGAATCGGAATGATACGAATCGGAATGGAACGAATCGGAATGTTCCGAATCGG
	AATGTATCGAATCGGAATGTGACGAATCGGAATGCGGC 3′
WC	3′ GCGAGCTTAGCCTTACCTAGCTTAGCCTTACTATGCTTAGCCTTACCTTGCTTAGCCTTACAAGGCTTAGCCTT
	ACATAGCTTAGCCTTACACTGCTTAGCCTTACGCCG 5′

**Table 11:** Checking for *φ*_2_ and *φ*_3_: the encoding rules of input strings.

Object of code	DNA code
Initiator sequence	*I*_1_ = 5′ CGCT 3′
Spacer sequence	*X*_0_ = 5′ CGA 3′, *X*_1_ = 5′ ATC 3′, *X*_2_ = 5′ GGA 3′, *X*_3_ = 5′ ATG 3′
Terminator sequence	*I*_2_ = 5′ CGGC 3′
Atomic proposition	*p*_1_ = 5′ GAT 3′, *p*_2_ = 5′ GAA 3′, *q*_1_ = 5′ ATA 3′, *q*_2_ = 5′ TTC 3′, *p* = 5′ TAT 3′, *q* = 5′ TGA 3′

**Table 12:** Checking for *φ*_2_ and *φ*_3_: the encoding of FSA A_5_ of formula, where sto() means WC.

Object of code	Abbreviated transition rule	DNA code
Initial state *s*_0_	None	3′ sto(*I*_1_ *X*_0_) 5′ = 3′ GCGA GCT 5′
Acceptance state *s*_2_	None	3′ sto(*X*_3_ *I*_2_) 5′ = 3′ TAC GCCG 5′
Transition rule *t*(*s*_0_, *p*_1_) = *s*_0_	*t*0*p*_1_0	3′ sto(*X*_1_*X*_2_*X*_3_ *p*_1_ *X*_0_) 5′ = 3′ TAG CCT TAC CTA GCT 5′
Transition rule *t*(*s*_0_, *p*) = *s*_0_	*t*0*p*0	3′ sto(*X*_1_*X*_2_*X*_3_ *p* *X*_0_) 5′ = 3′ TAG CCT TAC ATA GCT 5′
Transition rule *t*(*s*_0_, *q*_1_) = *s*_1_	*t*0*q*_1_1	3′ sto(*X*_1_*X*_2_*X*_3_ *q*_1_ *X*_0_*X*_1_) 5′ = 3′ TAG CCT TAC TAT GCT TAG 5′
Transition rule *t*(*s*_0_, *q*) = *s*_2_	*t*0*q*2	3′ sto(*X*_1_*X*_2_*X*_3_ *q* *X*_0_*X*_1_*X*_2_) 5′ = 3′ TAG CCT TAC ACT GCT TAG CCT 5′
Transition rule *t*(*s*_1_, *p*_2_) = *s*_1_	*t*1*p*_2_1	3′ sto(*X*_2_*X*_3_ *p*_2_ *X*_0_*X*_1_) 5′ = 3′ CCT TAC CTT GCT TAG 5′
Transition rule *t*(*s*_1_, *q*_2_) = *s*_2_	*t*1*q*_2_2	3′ sto(*X*_2_*X*_3_ *q*_2_ *X*_0_*X*_1_*X*_2_) 5′ = 3′ CCT TAC AAG GCT TAG CCT 5′
Transition rule *t*(*s*_2_, *p*) = *s*_0_	*t*2*p*0	3′ sto(*X*_3_ *p* *X*_0_) 5′ = 3′ TAC ATA GCT 5′
Transition rule *t*(*s*_2_, *q*) = *s*_2_	*t*2*q*2	3′ sto(*X*_3_ *q* *X*_0_*X*_1_*X*_2_) 5′ = 3′ TAC ACT GCT TAG CCT 5′

**Table 13:** The runs of the system *M*_2_.

Path	DNA code of the path or sequence of nodes (atomic propositions) crossed by the path
Code of path 1	CGCT CGAATCGGAATG GAT CGAATCGGAATG GAT | ATA CGAATCGGAATG GAA CGAATCGGAATG TTC CGAATCGGAATG CGGC
Sequence of nodes crossed by path 1	0,1, 2,3 (*p*_1_, *p*_1_ | *q*_1_, *p*_2_, *q*_2_)
Code of path *k*	CGCT CGAATCGGAATG (GAT CGAATCGGAATG GAT | ATA CGAATCGGAATG)^k^ GAA CGAATCGGAATG TTC CGAATCGGAATG CGGC
Sequence of nodes crossed by path *k*	(0,1)^*k*^, 2,3

**Table 14:** The results: checking for *φ*_2_ in the different paths of *M*_2_ (whether or not the path satisfies *φ*_2_).

Formula	Path 1	Path *k*, where 11 > *k* > 1	Path 11	Does *M*_2_ satisfy *φ*_2_?
*φ*_2_	Yes	Yes	Yes	Yes

**Table 15:** The runs of the system *M*_3_.

Path	DNA code of the path or sequence of nodes (atomic propositions) crossed by the path
Code of path 1	CGCT CGAATCGGAATG TAT CGAATCGGAATG TGA CGAATCGGAATG CGGC
Sequence of nodes crossed by path 1	0,1 (*p*, *q*)
Code of path *k*	CGCT CGAATCGGAATG (TAT CGAATCGGAATG TGA CGAATCGGAATG)^*k*^ CGGC
Sequence of nodes crossed by path *k*	(0,1)^*k*^

**Table 16:** The results: checking for *φ*_3_ in the different paths of *M*_3_ (whether or not the path satisfies *φ*_3_).

Formula	Path 1	Path *k*, where 6 > *k* > 1	Path 6	Does *M*_3_ satisfy *φ*_3_?
*φ*_3_	Yes	Yes	Yes	Yes

**Table 17:** Checking for *φ*_4_: the designed encoding sequence.

Code	5′ GCAGTCGGCGCATCATGTGGTCTTTGCATGGACGTAGTGATCGGCGCATCATGTGGTCTTTGCATGGACGTAAT
	CCTCGGCGCATCATGTGGTCTTTGCATGGACGTAAACGTCGGCGCATCATGTGGTCTTTGCATGGACGTAGGGAT
	CGGCGCATCATGTGGTCTTTGCATGGACGTAAACCCCGCCAAATTACATATGACCGACG 3′

WC	3′ CGTCAGCCGCGTAGTACACCAGAAACGTACCTGCATCACTAGCCGCGTAGTACACCAGAAACGTACCTGCATT
	AGGAGCCGCGTAGTACACCAGAAACGTACCTGCATTTGCAGCCGCGTAGTACACCAGAAACGTACCTGCATCCC
	TAGCCGCGTAGTACACCAGAAACGTACCTGCATTTGGGGCGGTTTAATGTATACTGGCTGC 5′

**Table 18:** Checking for *φ*_4_: the encoding rules of input strings.

Object of code	DNA code (they are all in 5′-3′ direction from left to right)
Initiator sequence	*I*_1_ = GCAG
Spacer sequence	*X*_0_ = TCGG, *X*_1_ = CGCA, *X*_2_ = TCAT, *X*_3_ = GTGG, *X*_4_ = TCTT, *X*_5_ = TGCA, *X*_6_ = TGGA, *X*_7_ = CGTA
Terminator sequence	*I*_2_ = AACC
Atomic proposition	*p*_1_ = CCGC, *q*_1_ = ATCC, *p*_2_ = CAAA, *q*_2_ = GGGA, *p*_3_ = TTAC, *q*_3_ = ATAT *m*_1_ = *p*_1_∧*p*_3_ = GTGA, *m*_2_ = *q*_1_∧*p*_3_ = GACC, *m*_3_ = *p*_2_∧*q*_3_ = AACG, *m*_4_ = *q*_2_∧*q*_3_ = GACG

**Table 19:** Checking for *φ*_4_: the encoding of FSA A_4_ of formula, where sto() means WC.

Object of code	Abbreviated transition rule	DNA code (they are all in 5′-3′ direction from left to right)
Initial state *s*_0_	None	sto(*I*_1_ *X*_0_) = CGTC AGCC
Acceptance state *s*_4_	None	sto(*X*_5_*X*_6_*X*_7_ *I*_2_) = ACGT ACCT GCAT TTGG
Transition rule *t*(*s*_0_, *m*_1_) = *s*_1_	*t*0*m*_1_1	sto(*X*_1_*X*_2_*X*_3_*X*_4_*X*_5_*X*_6_*X*_7_ *m*_1_ *X*_0_*X*_1_) = GCGT AGTA CACC AGAA ACGT ACCT GCAT CACT AGCC GCGT
Transition rule *t*(*s*_0_, *m*_2_) = *s*_2_	*t*0*m*_2_2	sto(*X*_1_*X*_2_*X*_3_*X*_4_*X*_5_*X*_6_*X*_7_ *m*_2_ *X*_0_*X*_1_*X*_2_) = GCGT AGTA CACC AGAA ACGT ACCT GCAT CTGG AGCC GCGT AGTA
Transition rule *t*(*s*_1_, *p*_1_) = *s*_1_	*t*1*p*_1_1	sto(*X*_2_*X*_3_*X*_4_*X*_5_*X*_6_*X*_7_ *p*_1_ *X*_0_*X*_1_) = AGTA CACC AGAA ACGT ACCT GCAT GGCG AGCC GCGT
Transition rule *t*(*s*_1_, *q*_1_) = *s*_2_	*t*1*q*_1_2	sto(*X*_2_*X*_3_*X*_4_*X*_5_*X*_6_*X*_7_ *q*_1_ *X*_0_*X*_1_*X*_2_) = AGTA CACC AGAA ACGT ACCT GCAT TAGG AGCC GCGT AGTA
Transition rule *t*(*s*_2_, *m*_3_) = *s*_3_	*t*2*m*_3_3	sto(*X*_3_*X*_4_*X*_5_*X*_6_*X*_7_ *m*_3_ *X*_0_*X*_1_*X*_2_*X*_3_) = CACC AGAA ACGT ACCT GCAT TTGC AGCC GCGT AGTA CACC
Transition rule *t*(*s*_2_, *m*_4_) = *s*_4_	*t*2*m*_4_4	sto(*X*_3_*X*_4_*X*_5_*X*_6_*X*_7_ *m*_4_ *X*_0_*X*_1_*X*_2_*X*_3_*X*_4_) = CACC AGAA ACGT ACCT GCAT CTGC AGCC GCGT AGTA CACC AGAA
Transition rule *t*(*s*_3_, *p*_2_) = *s*_3_	*t*3*p*_2_3	sto(*X*_4_*X*_5_*X*_6_*X*_7_ *p*_2_ *X*_0_*X*_1_*X*_2_*X*_3_) = AGAA ACGT ACCT GCAT GTTT AGCC GCGT AGTA CACC
Transition rule *t*(*s*_3_, *q*_2_) = *s*_4_	*t*3*q*_2_4	sto(*X*_4_*X*_5_*X*_6_*X*_7_ *q*_2_ *X*_0_*X*_1_*X*_2_*X*_3_*X*_4_) = AGAA ACGT ACCT GCAT CCCT AGCC GCGT AGTA CACC AGAA

**Table 20:** The runs of the system *M*_2_.

Path	DNA code of the path or sequence of nodes (atomic propositions) crossed by the path
Code of path 1	GCAG TCGGCGCATCATGTGGTCTTTGCATGGACGTA CCGC | GTGA TCGGCGCATCATGTGGTCTTTGCATGGACGTA CCGC | ATCC TCGGCGCATCATGTGGTCTTTGCATGGACGTA CAAA TCGGCGCATCATGTGGTCTTTGCATGGACGTA GGGA TCGGCGCATCATGTGGTCTTTGCATGGACGTA AACC
Sequence of nodes crossed by path 1	0,1, 2,3 (only the atomic propositions that occur in the transition rules are retained: *p*_1_ | *m*_1_, *p*_1_ | *q*_1_, *p*_2_, *q*_2_)
Code of path *k*	GCAG TCGGCGCATCATGTGGTCTTTGCATGGACGTA (CCGC | GTGA TCGGCGCATCATGTGGTCTTTGCATGGACGTA CCGC | ATCC TCGGCGCATCATGTGGTCTTTGCATGGACGTA)^k^ CAAA TCGGCGCATCATGTGGTCTTTGCATGGACGTA GGGA TCGGCGCATCATGTGGTCTTTGCATGGACGTA AACC
Sequence of nodes crossed by path *k*	(0,1)^*k*^, 2,3

**Table 21:** A comparison of power among the various DNA model checking methods (can the method conduct DNA model checking for a given formula?).

Logic	Basic formula	Method in [53]	Method in [55]	Method in [54]	The new method	What the new method can do
LTL	*p*U*q*	No	Yes	Yes	No	—
	F*p*	No	Yes	The method can be used to check. However, it is not practical to check due to the limitation of the code	No	—
	G*p*	No	Yes	The method can be used to check. However, it is not practical to check due to the limitation of the code	No	—
	X*p*	No	Yes	No	No	—

CTL	A*p*U*q*	No	No	No	Yes	A combination of Algorithm 1 and the experiments in [55]
	AF*p*	No	No	No	Yes	
	AG*p*	No	No	No	Yes	
	AX*p*	No	No	No	Yes	
	E*p*U*q*	No	No	No	Yes	The experiments for *φ*_1_ in Section 4.1
	EF*p*	Yes	No	No	Yes	A combination of Algorithm 2 and the experiments in [55]
	EG*p*	No	No	No	Yes	
	EX*p*	No	No	No	Yes	

ITL	(*p*_1_U*q*_1_); (*p*_2_U*q*_2_)	No	No	No	Yes	The experiments for *φ*_2_ in Section 4.2
	(*p*U*q*)^*∗*^	No	No	No	Yes	The experiments for *φ*_3_ in Section 4.2

PTL	((*p*_1_U*q*_1_), (*p*_2_U*q*_2_)) prj (*p*_3_∧X*q*_3_)	No	No	No	Yes	The experiments for *φ*_4_ in Section 4.3

## Appendix

Sections 2.1, 2.2, 2.3, and 2.4 give the intuitive meaning of the four temporal logic types. The Appendix gives the formal descriptions of these temporal logic types.

### A. Linear Temporary Logic (LTL)

Definition A.1 A.1 (syntax of propositional LTL [43], LTL for short). Let AP be a set of atomic propositions; then,

- for all *p* ∈ AP, *p* is a LTL formula;
- if *φ* is a LTL formula, then ¬*φ* is a LTL formula;
- if *φ* and *ψ* are LTL formulas, then *φ*∨*ψ* is a LTL formula;
- if *φ* is a LTL formula, then X*φ* is a LTL formula;
- if *φ* and *ψ* are LTL formulas, then *φ*U*ψ* is a LTL formula.

Definition A.2 A.2 (derived LTL formulas). F*φ* = *true*U*φ*, G*φ* = ¬F¬*φ*, *φ*∧*ψ* = ¬(¬*φ*∨¬*ψ*).

Definition A.3 A.3 (model for LTL). A LTL model is a triple M = (*S*, *R*, *LABEL*), where

- *S* is a nonempty denumerable set of states,
- *R* : *S* → *S* assigns to *s* ∈ *S* its unique successor state *R*(*s*),
- *LABEL* : *S* → 2^AP^ assigns to each state *s* ∈ *S* the atomic propositions *LABEL*(*s*) that are valid in *s*.

Definition A.4 A.4 (semantic of LTL). Let *p* ∈ AP be an atomic proposition, M = (*S*, *R*, *LABEL*) a LTL model, *s* ∈ *S*, and *φ*, *ψ* PLTL formulas; the satisfaction relation ⊨ is defined by

- *s*⊨*p* if and only if (iff, for short) *p* ∈ *LABEL*(*s*),
- *s*⊨¬*φ* if and only if ¬(*s*⊨*φ*),
- *s*⊨*φ*∨*ψ* if and only if (*s*⊨*φ*)∨(*s*⊨*ψ*),
- *s*⊨X*φ* if and only if *R*(*s*)⊨*φ*,
- *s*⊨*φ*U*ψ* if and only if ∃*j* ≥ 0. *R*^*j*^(*s*)⊨*ψ*∧(∀0 ≤ *k* < *j*. *R*^*k*^(*s*)⊨*φ*).

Here, *R*^0^(*s*)⊨*s*, and *R*^*n*+1^(*s*) = *R*^*n*^(*R*(*s*)) for any *n* ≥ 0, where *R*(*s*) = *s*′ and the state *s*′ is called a direct successor of *s*.

### B. Interval Temporal Logic (ITL)

Definition B.1 B.1 (syntax of propositional ITL [50], ITL for short). (i) For all *p* ∈ AP, *p* is an ITL formula. (ii) If *φ*, *ψ* are ITL formulas, so are the constructs ¬*φ*, *φ*∨*ψ*, X*φ*, *φ*; *ψ*, *φ*^*∗*^.

Definition B.2 B.2. An interval of states is defined and also denoted as *σ* = 〈*s*_0_, *s*_1_,…, *s*_*i*_,…〉, where *s*_*i*_ is a state.

Definition B.3 B.3. An interpretation is a quadruple *I* = (*σ*, *i*, *k*, *j*), where *σ* is a sequence of states over 〈*s*_*i*_,…, *s*_*k*_,…, *s*_*j*_〉, *i*, *k*, *j* ∈ *N*, and *s*_*k*_ is the current state. We use the notation len(*σ*) ⫤  *σ*⊨*j* − *i* for the number of states in interval.

Definition B.4 B.4 (semantic of ITL). Let *c* ∈ *N* and *s*_*p*_(*k*) be the true value of *p* ∈ AP in state *s*_*k*_. The satisfaction relation is inductively defined as follows:

1. *I*⊨*p* iff *s*_*p*_(*k*) = true,
2. *I*⊨¬*φ* iff ¬(*I*⊨*φ*),
3. *I*⊨*φ*_1_∨*φ*_2_ iff *I*⊨*φ*_1_ or *I*⊨*φ*_2_,
4. *I*⊨*skip* iff len(*σ*) = 1,
5. *I*⊨X*φ* iff (*σ*, *i*, *k* + 1, *j*)⊨*φ*,
6. *I*⊨*φ*_1_; *φ*_2_ iff ∃*r*, *k* ≤ *r* ≤ *j*, such that (*σ*, *i*, *k*, *r*)⊨*φ*_1_ and (*σ*, *i*, *k*, *r*)⊨*φ*_2_,
7. *I*⊨*φ*^*∗*^ iff (i) there exist finitely many *r*_0_,…, *r*_*n*_ ∈ *N*_*ω*_, such that *k* = *r*_0_ ≤ *r*_1_ ≤ ⋯≤*r*_*n*−1_ ≤ *r*_*n*_ = *j*, (*σ*, *i*, *k*, *r*_0_)⊨*φ*, and for every 1 ≤ *l* ≤ *n*, (*σ*, *r*_*l*−1_, *r*_*l*−1_, *r*_*l*_)⊨*φ*; (ii) or *k* = *j*.

Definition B.5 B.5 (derived ITL formulas). F*φ* = *true*; *φ*, G*φ* = ¬F¬*φ*, *φ*∧*ψ* = ¬(¬*φ*∨¬*ψ*), *φ*_1_||*φ*_2_ = *φ*_1_∧(*φ*_2_; *true*)∨*φ*_2_∧(*φ*_1_; *true*).

### C. Computational Tree Logic (CTL)

Definition C.1 C.1 (syntax of propositional CTL [49], CTL for short). Given a set of basic propositions *P*, a CTL formula *ϕ* on *P* is any recursive combination of the propositions of *P* through the Boolean connectives* not* (¬*ϕ*),* and* (*ϕ*_1_∧*ϕ*_2_), and through the temporal operators* existential until* (E[*ϕ*_1_U*ϕ*_2_])* universal until* (A[*ϕ*_1_U*ϕ*_2_]):

(C.1) $$\begin{array}{c} \phi =p\left|\;\neg \phi_{1}\;\right|\phi_{1}\wedge \phi_{2}\left|\;E\left[\;\phi_{1}U\phi_{2}\;\right]\;\right|A\left[\;\phi_{1}U\phi_{2}\;\right] \end{array}$$

with *p* ∈ *P*.

Definition C.2 C.2 (semantic of CTL). Given a state transition system *S* and a state *s*_0_ ∈ *S*, a CTL formula *ϕ* is interpreted on *S* according to the satisfaction relation *s*_0_⊨*ϕ* (read *s*_0_ satisfies *ϕ*) defined by the following clauses:

- *s*_0_⊨*p* iff the proposition *p* holds in *s*_0_;
- *s*_0_⊨¬*ϕ*_1_ iff *ϕ*_1_is not satisfied in *s*_0_;
- *s*_0_⊨*ϕ*_1_∧*ϕ*_2_ iff both *ϕ*_1_ and *ϕ*_2_ are satisfied in *s*_0_;
- *s*_0_⊨E[*ϕ*_1_U*ϕ*_2_] iff there exists a path starting from *s*_0_ which reaches a future state in which *ϕ*_2_ is satisfied and such that *ϕ*_1_ is satisfied in every intermediate state;
- *s*_0_⊨A[*ϕ*_1_U*ϕ*_2_] iff along every path starting from *s*_0_ a future state is eventually reached in which *ϕ*_2_ is satisfied, and *ϕ*_1_ is satisfied in all the intermediate states.

Definition C.3 C.3 (short hands). Further operators can be defined as short hands by combining Boolean connectives and temporal operators.

(i) Boolean connectives such as* or* (∨) and* implies* (→), with their usual meaning, can be derived as
  (C.2) ϕ1∨ϕ2=¬¬ϕ1∧¬ϕ2,ϕ1⟶ϕ2=¬ϕ1∧¬ϕ2.
(ii) Temporal operators* eventually* (F) and* always* (G), with existential and universal path quantification, are derived as follows:
  (C.3) EFϕ2==EtrueUϕ2,AFϕ2==AtrueUϕ2,EGϕ1==¬AF¬ϕ1,AGϕ1==¬EF¬ϕ1.

### D. Projection Temporal Logic (PTL)

Definition D.1 D.1 (syntax of propositional PTL [52], PTL for short). Let *p* and *q* be atomic propositions. The formula *P* of PPTL is given by the following grammar:

(D.1) $$\begin{array}{c} P⩴p\left|\;XP\;\right|\neg P\left|P_{1}\vee P_{2}\right|\left(P_{1},\ldots ,P_{m}\right)prj\;\;P, \end{array}$$

where *p* ∈ *Prop*, *P*_1_,…, *P*_*m*_ and *P* are all well-formed PPTL formulas.

Definition D.2 D.2 (semantic of PTL).  
*(1) States*. Following the definition of Kripke's structure, we define a state *s* over *Prop* to be a mapping from *Prop* to *B* = {*true*, *false*}, *s* : *Prop* → *B*. We will use *s*[*p*] to denote the valuation of *p* at state *s*.
*(2) Intervals*. An interval *σ* is a nonempty sequence of states, which can be finite or infinite. The length, |*σ*|, of *σ* is *ω* if *σ* is infinite, with the number of states minus 1 if *σ* is finite. To have a uniform notation for both finite and infinite intervals, we will use extended integers as indices. That is, we consider the set *N*_0_ of nonnegative integers and *ω*, *N*_*ω*_ = *N*_0_ ∪ {*ω*}, and extend the comparison operators, = , <, ≤, to *N*_*ω*_ by considering *ω* = *ω*, and for all *i* ∈ *N*_0_, *i* < *ω*. Moreover, we define ⪯ as ≤−{(*ω*, *ω*)}. To simplify definitions, we will denote *σ* by 〈*s*_0_,…, *s*_|*σ*|_〉, where *s*_|*σ*|_ is undefined if *σ* is infinite. With such a notation, *σ*_(*i*,…,*j*)_  0 ≤ *i*⪯*j*≤|*σ*| denotes the subinterval 〈*s*_*i*_,…, *s*_*j*_〉 and *σ*^(*k*)^  (0 ≤ *k*⪯|*σ*|) denotes 〈*s*_*k*_,…, *s*_|*σ*|_〉. The concatenation of a finite *σ* with another interval (or empty string) *σ*′ is denoted by *σ* · *σ*′. Let *σ* = 〈*s*_0_, *s*_1_,…, *s*_|*σ*|_〉 be an interval and *r*_1_,…, *r*_*h*_ be integers (*h* ≥ 1) such that 0 ≤ *r*_1_ ≤ *r*_2_ ≤ ⋯≤*r*_*h*_⪯|*σ*|. The projection of *σ* onto *r*_1_,…, *r*_*h*_ is the interval (namely, projected interval)

(D.2) $$\begin{array}{c} \sigma ↓\left(\;r_{1},\ldots ,r_{h}\;\right)=\langle \;s_{t1},s_{t2},\ldots ,s_{tl}\;\rangle , \end{array}$$

where *t*_1_,…, *t*_*l*_ is obtained from *r*_1_,…, *r*_*h*_ by deleting all duplicates. That is, *t*_1_,…, *t*_*l*_ is the longest strictly increasing subsequence of *r*_1_,…, *r*_*h*_. For instance,

(D.3) $$\begin{array}{c} \langle \;s_{0},s_{1},s_{2},s_{3},s_{4}\;\rangle ↓\left(\;0,0,2,2,2,3\;\right)=\langle \;s_{0},s_{2},s_{3}\;\rangle . \end{array}$$

This is convenient to define an interval obtained by taking the endpoints (rendezvous points) of the intervals over which *P*_1_,…, *P*_*m*_ are interpreted in the projection construct.
*(3) Interpretations and Satisfaction Relation*. An interpretation is a triple *I* = (*σ*, *k*, *j*), where *σ* is an interval, *k* is an integer, and *j* is an integer or *ω* such that *k*⪯*j*≤|*σ*|. We use the notation (*σ*, *k*, *j*)⊨*P* to denote that formula *P* is interpreted and satisfied over the subinterval 〈*s*_*k*_,…, *s*_*j*_〉 of *σ* with the current state being *s*_*k*_. The satisfaction relation (⊨) is inductively defined as follows:

1. *I*⊨*p* iff *s*_*k*_[*p*] = *true*, for any given proposition *p*;
2. *I*⊨¬*P* iff *I*⊭*P*;
3. *I*⊨*P*∨*Q* iff *I*⊨*P* or *I*⊨*Q*;
4. *I*⊨X*P* iff *k* < *j* and (*σ*, *k* + 1, *j*)⊨*P*;
5. *I*⊨(*P*_1_,…, *P*_*m*_)  *prj*  *Q* if there exist integers *k* = *r*_0_ ≤ *r*_1_ ≤ ⋯≤*r*_*m*_ ≤ *j* such that (*σ*, *r*_0_, *r*_1_)⊨*P*_1_, (*σ*, *r*_*l*−1_, *r*_*l*_)⊨*P*_*l*_, 1 < *l* ≤ *m*, and (*σ*′, 0, |*σ*′|)⊨*Q* for one of the following *σ*′:
  - *r*_*m*_ < *j* and *σ*′ = *σ* ↓ (*r*_0_,…, *r*_*m*_) · *σ*_(*r*_*m*_+1⋯*j*)_;
  - *r*_*m*_ = *j* and *σ*′ = *σ* ↓ (*r*_0_,…, *r*_*h*_) for some 0 ≤ *h* ≤ *m*.
